# The great multivariate time series classification bake off: a review and experimental evaluation of recent algorithmic advances

**DOI:** 10.1007/s10618-020-00727-3

**Published:** 2020-12-18

**Authors:** Alejandro Pasos Ruiz, Michael Flynn, James Large, Matthew Middlehurst, Anthony Bagnall

**Affiliations:** School of Computing Sciences, University of East Anglia, Norwich, UK

**Keywords:** Time series classification, Evaluating classifiers, Multivariate time series, UEA archive

## Abstract

Time Series Classification (TSC) involves building predictive models for a discrete target variable from ordered, real valued, attributes. Over recent years, a new set of TSC algorithms have been developed which have made significant improvement over the previous state of the art. The main focus has been on univariate TSC, i.e. the problem where each case has a single series and a class label. In reality, it is more common to encounter multivariate TSC (MTSC) problems where the time series for a single case has multiple dimensions. Despite this, much less consideration has been given to MTSC than the univariate case. The UCR archive has provided a valuable resource for univariate TSC, and the lack of a standard set of test problems may explain why there has been less focus on MTSC. The UEA archive of 30 MTSC problems released in 2018 has made comparison of algorithms easier. We review recently proposed bespoke MTSC algorithms based on deep learning, shapelets and bag of words approaches. If an algorithm cannot naturally handle multivariate data, the simplest approach to adapt a univariate classifier to MTSC is to ensemble it over the multivariate dimensions. We compare the bespoke algorithms to these dimension independent approaches on the 26 of the 30 MTSC archive problems where the data are all of equal length. We demonstrate that four classifiers are significantly more accurate than the benchmark dynamic time warping algorithm and that one of these recently proposed classifiers, ROCKET, achieves significant improvement on the archive datasets in at least an order of magnitude less time than the other three.

## Introduction

Time series classification (TSC) is a form of machine learning where the features of the input vector are real valued and ordered. This scenario adds a layer of complexity to the problem, as important characteristics of the data can be missed by traditional algorithms. Over recent years, a new set of TSC algorithms have been developed which have made significant improvement over the previous state of the art (Bagnall et al. 2017).

The main focus has been on univariate TSC, i.e. the problem where each case has a single series and a class label. In reality, it is more common to encounter multivariate TSC (MTSC) problems where the time series for a single case has multiple dimensions. Human activity recognition, diagnosis based on electrocardiogram (ECG), electroencephalogram (EEG) and Magnetoencephalography (MEG), and systems monitoring problems are all inherently multivariate. Despite this, much less consideration has been given to MTSC than the univariate case. The UCR archive has provided a valuable resource for univariate TSC, and its existence may explain the growth of algorithm development for this task. Until recently, there were few resources for MTSC. An archive of 30 MTSC problems released in Bagnall et al. (2018) has made comparison of algorithms easier and will we hope spur further research in this field. We compare recently proposed bespoke MTSC algorithms to simple adaptations of univariate approaches on the 26 equal length problems in the UEA MTSC archive. We find that dynamic time warping (DTW) is still hard to beat in MTSC, but that four algorithms are significantly more accurate than this benchmark on this archive. It is dangerous to infer too much from results achieved on 26 problems collected in an arbitrary way across a wide range of problem domains. Nevertheless, some advice for practitioners for a starting point in an analysis is always helpful. We conclude that one recently published algorithm, ROCKET (Dempster et al. 2020), is our recommended choice due to high overall accuracy and remarkably fast training time.

We provide an overview of MTSC and the classifiers we evaluate in Sect. 2, and the datasets used in Sect. 3. The experimental and evaluation procedures are defined in Sect. 4, along with the results of preparatory experiments into the data and benchmark classifier definitions used throughout the main evaluation. We present an analysis of the results in Sect. 5. Conclusions are drawn in Sect. 6. Comprehensive results and a guide to reproducing them are provided on the accompanying website.^1^

## Background

In univariate time series classification, an instance is a pair $\{{\varvec{x}}, y\}$ with *m* observations $(x_1,\ldots , x_m)$ (the time series) and discrete class variable *y* with *c* possible values. A classifier is a function or mapping from the space of possible inputs to a probability distribution over the class variable values. In MTSC, the time series is a list of vectors over *d* dimensions and *m* observations, ${\varvec{X}}=<{\varvec{x_1}}, \ldots {\varvec{x_d}}>$, where ${\varvec{x_k}}=(x_{1,k},x_{2,k},\ldots ,x_{m,k})$. We denote the *j*th observation of the *i*th case of dimension *k* as the scalar $x_{i,j,k}$.

The core additional complexity for MTSC is that discriminatory features may be in the interactions between dimensions, not just in the autocorrelation within an individual series. In univariate TSC features may or may not be phase-dependent, while in MTSC features may or may not be simultaneously dimension-dependent. Further, the sheer volume of data may obscure discriminatory features. Algorithms for MTSC can be categorised in similar ways as algorithms for univariate TSC on whether they are based on: distance measures; shapelets; histograms over a dictionary; interval summarising; or deep learning/neural networks.

We have attempted to include a variety of algorithms in our evaluation: those of different algorithm archetypes, some bespoke to the multivariate case, and others that would be recognised from the univariate case. Ultimately though, the selection criteria for classifiers was largely practical. We had to have access to the source code and be be able to run the algorithm.

Distance based approaches are mainly based on dynamic time warping (DTW). DTW has been a popular benchmark in TSC, at one time being the ‘gold standard’. While it can now be beaten on average across arbitrary datasets it is still often used as a baseline for comparison. Three proposed approaches to generalising dynamic time warping to the multivariate case from Shokoohi-Yekta et al. (2017) are described in Sect. 2.1. Adopting DTW as an initial benchmark for MTSC seems a clear choice.

Another obvious benchmark is to adapt univariate algorithms to the multivariate case and leverage their relative advancement and familiarity. We can achieve this simply by ensembling over dimensions and implicitly assume independence between them. We elaborate on this in Sect. 2.2. In Sects. 2.3 to 2.8 we cover the range of classifiers included in our study that are designed for the multivariate case, or have been non-trivially converted from the univariate case.

### Dynamic time warping

One of the most popular approaches for TSC is to use a 1-nearest neighbourhood classifier in conjunction with a bespoke distance function that compensates for possible confounding offset by allowing some realignment of the series. Dynamic time warping (DTW) is the most popular distance function for this purpose. DTW can be used with unequal series, but for simplicity we describe it with reference to equal length series. In DTW, the distance between two series of equal length $\mathbf{a }=(a_1,a_2,\ldots , a_m)$ and $\mathbf{b }=(b_1,b_2,\ldots , b_m)$ is calculated following these steps:

1. *M* is a $m \times m$ matrix where $M_{i,j}=(a_i-b_j)^2$
2. A warping path $P=((e_1,f_1),(e_2,f_2),\ldots ,(e_s,f_s))$ is a contiguous set of matrix indexes from *M*, subject to the following constraints
  $$(e_1,f_1)=(1,1)$$
  $$(e_s,f_s)=(m,m)$$
  $$0 \le e_{i+1} - e_i \le 1$$ for all $$i<m$$
  $$0 \le f_{i+1} - f_i \le 1$$ for all $$i<m$$
3. Let $p_i=M_{e_i,f_i}$, be the distance for a path is $D_p=\sum _{i=1}^m p_i$
4. There are many warping paths but we are interested in finding one of the paths that minimizes the accumulative distance $P^*=\min _{p \in P} D_p(a,b)$
5. The optimal distance is obtained by solving the following recurrence relation
  $$\begin{aligned} DTW(i,j)=M_{i,j}+ min{\left\{ \begin{array}{ll} DTW(i-1, j).\\ DTW(i, j-1).\\ DTW(i-1, j-1), \end{array}\right. } \end{aligned}$$
  and the final distance is *DTW*(*m*, *m*).

There are several improvements to DTW to make it faster, such as, adding a parameter *r* that limits deviation from the diagonal (warping window). Our interest lies primarily in how best to use DTW for MTSC. There are two obvious strategies for using DTW for multivariate problems, defined in Shokoohi-Yekta et al. (2017) as the independent and dependent approaches.

#### Independent warping ($DTW_I$)

The independent strategy treats each dimension independently, has a different pointwise distance matrix *M* for each dimension, then sums the resulting DTW distances.

$$\begin{aligned} DTW_I({\varvec{x_a}},{\varvec{x_b}}) = \sum _{k=1}^d DTW({\varvec{x_{a,k}}},{\varvec{x_{b,k}}}) \end{aligned}$$

#### Dependent warping ($DTW_D$)

Dependent warping assumes that the correct warping is the same across all dimensions. For handling this case, the matrix $M_{i,j}$ is redefined not as the distance between two points on a single series but as the Euclidean distance between the two vectors that represent all the dimensions. Thus warping occurs over all dimensions simultaneously and the time point distance between steps *i* and *j* is given by

$$\begin{aligned} M_{i,j}({\varvec{x_a}},{\varvec{x_b}})=\sum _{k=1}^d (x_{a,i,k}-x_{b,j,k})^2 \end{aligned}$$

#### Adaptive warping ($DTW_A$)

Shokoohi-Yekta et al. (2017) discuss the idea of selecting between independent and dependent dynamic time warping. They proposed an adaptive solution, where the decision about which distance to use is based on a threshold found from the training data. This decision is made instance by instance basis based on the score function *S*(*x*)

$$\begin{aligned} S(x)=\frac{NN_{DTW_D}(x)}{NN_{DTW_I}(x)} \end{aligned}$$

where $NN_C$ is the distance of the nearest neighbour to *x* using the distance function *C*. The final nearest neighbour to use for classification is based on the threshold *T*

$$\begin{aligned} NN_{DTW_A}(x)={\left\{ \begin{array}{ll} NN_{DTW_I}(x) &{}\text{ if } S(X)>T .\\ NN_{DTW_D}(x)&{}\text{ if } S(X) \le T. \end{array}\right. } \end{aligned}$$

The threshold value *T* is calculated from training data using cross validation. Each instance *x* is classified using $DTW_I$ and $DTW_D$. If the instance is classified correctly using $DTW_I$ but incorrectly on $DTW_D$, then is added to the set *iSuccess*. Otherwise, if it is classified correctly on $DTW_D$ but incorrectly on $DTW_I$, then is added to the set *dSuccess*. On each set, the value stored is the score function defined in Eq. 2.1.3. The threshold is calculated using information gain on *iSuccess* and *dSuccess* data. Information gain calculates the split point where most of the *iSuccess* cases are on one side and the *dSuccess* on the other. Using this variation, each instance will use the distance function that maximises the probability of getting the correct classification and minimise the error $error(NN_{DTW_A}(x)\le min(NN_{DTW_D}(x),NN_{DTW_I}(x))$.

### Ensembles of univariate classifiers

One of the most straightforward techniques to adapt TSC algorithms to multivariate is to ensemble over models built on each dimension independently. This approach is a good baseline for assessing and contrasting bespoke MTSC classifiers which can model dimension dependencies. One of the most accurate approaches to univariate TSC is the Hierarchical Vote Collective of Transformation-based Ensembles (HIVE-COTE). The latest version, HIVE-COTE v1.0 (referred to as simply HIVE-COTE, Bagnall et al. 2020), combines Shapelet Transform Classifier, STC (Hills et al. 2014); Time Series Forest, TSF (Deng et al. 2013); Contractable Bag of Symbolic-Fourier Approximation Symbols, CBOSS (Middlehurst et al. 2019) and Random Interval Spectral Ensemble, RISE (Lines et al. 2018) using a weighted probabilistic ensemble (Large et al. 2019). The simplest way to build a multivariate HIVE-COTE is to build each component as an independent ensemble, then to combine the components in the usual way. To clarify, each component builds a separate classifier on every dimension, then combines the predictions from each dimension to produce a single probability distribution for each of STC, TSF, CBOSS and RISE.

### Generalized random shapelet forest (gRFS)

Shapelets (Ye and Keogh 2011) are discriminatory sub-series which are easily interpretable. Early shapelet algorithms enumerated all possible shapelets and hence scaled poorly. Karlsson et al. (2016) propose a shapelet based approach for MTSC. The algorithm uses randomly selected shapelets within a forest of decision trees, modelled on the random forest approach (Breiman 2001).
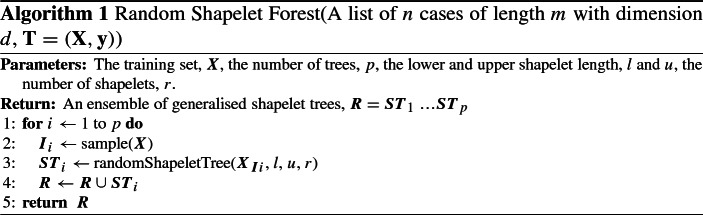


The Generalised Random Forest (gRFS) (illustrated in Algorithms 1 and 2) is an ensemble of weak learners in which *p* generalized trees are grown. In order to introduce variability amongst the constituent classifiers a bagging approach is employed. Algorithm 2 works as follows. At each node in a generalised tree a dimension, *k*, is randomly selected to proceed with (line 3). From this dimension *r* shapelets are selected from the training set *Z*. Each shapelet has a randomly selected length (line 5) between predefined upper and lower limits *u* and *l*. The shapelet selected at each node corresponds to that which produces the most favourable split (line 6). The quality of a shapelet is measured using information gain. The data is split by the information gain threshold of the selected shapelet and a tree recursively is recursively built (lines 8 and 9) until the stopping condition is met (line 1 and 2).
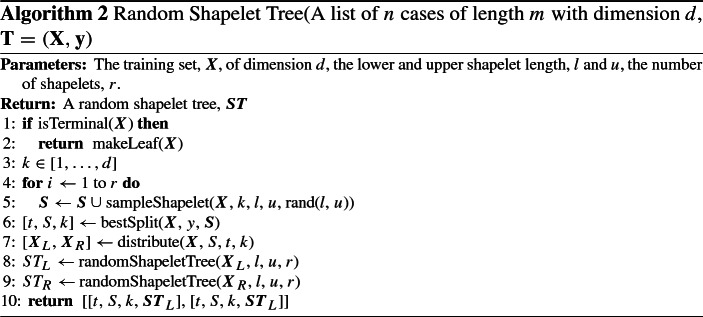


### WEASEL+MUSE

Originally a univariate time series classifier, Word Extraction for Time Series Classification, WEASEL (Schäfer and Leser 2017) was extended to include the Multivariate Unsupervised Symbols and Derivatives, MUSE (Schäfer and Leser 2018) stage for MTSC. Words in the form of unigrams and bigrams are extracted for all series and dimensions using a sliding window for a range of window lengths. These words are extracted using the Symbolic Fourier Approximation, SFA (Schäfer and Högqvist 2012) with equi-depth or equi-frequency binning. Words for the derivatives (differences between neighbouring points in the series) of each dimension are also taken and treated as additional dimensions. The words for each dimension and window length are concatenated into a single bag of words histogram for a series. As this process produces a lot of words with a presumed amount of redundancy and to filter out unproductive dimensions, a $\chi ^2$ test is used for feature selection. The remaining words are used to build a logistic regression classifier.

A 10-fold cross validation is performed to select parameters for the final WEASEL+MUSE model. These are the word length *l*, the binning method *b* and whether to normalise each window *p*. The WEASEL+MUSE build process is displayed in Algorithm 3. For simplicity, we will refer to this algorithm as just MUSE forthwith.
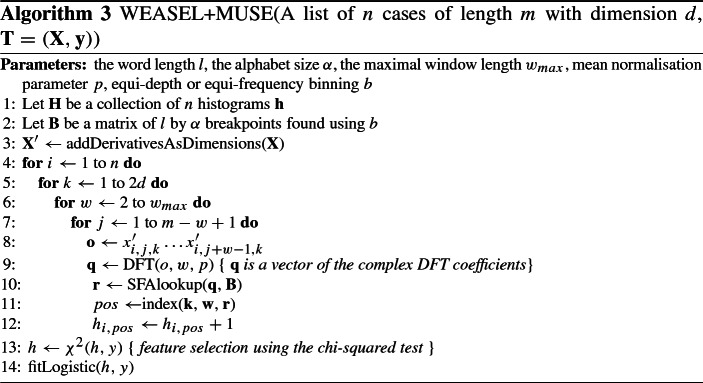


### Canonical interval forest (CIF)

The Canonical Interval Forest, CIF (Middlehurst et al. 2020) is an ensemble of time series tree (Deng et al. 2013) classifiers built using the Canonical Time-Series Characteristics, Catch22 (Lubba et al. 2019) features and simple summary statistics extracted from phase dependant intervals. The time series tree uses a simplistic tree structure, comparing all attributes at each node and performing no pruning. However, the tree introduces a novel tie breaking measure in the form of entrance gain. Catch22 is a set of 22 highly discriminative and low redundancy features extracted from the 7000+ time series features available in the Highly Comparative Time Series Analysis (hctsa) toolbox (Fulcher and Jones 2017).

To create a diverse ensemble, *a* summary features of the 25 available are randomly subsampled and *k* intervals of random length and start point are selected to build each tree. CIF was extended for MTSC by randomly selecting the dimension each interval is extracted from. The build process for the CIF ensemble is described in Algorithm 4.
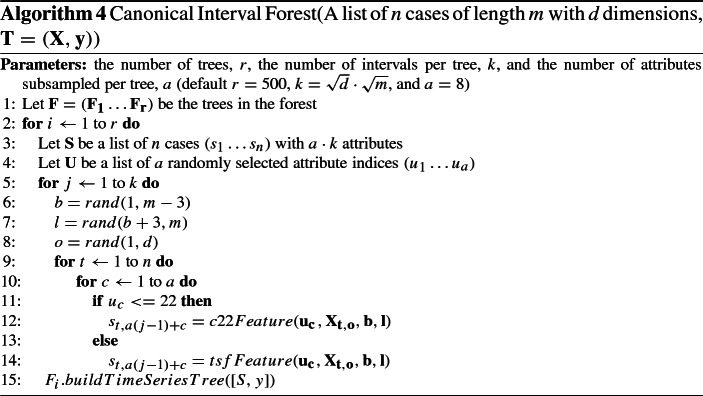


### The random convolutional kernel transform (ROCKET)

The Random Convolutional Kernel Transform, ROCKET (Dempster et al. 2020) uses a large number of random convolution kernels in conjunction with a linear classifier (ridge regression or logistic regression). Every kernel is applied to each instance. From the resulting feature maps, the maximum value and a novel feature, proportion of positive values (ppv), is returned.

For each of the 10,000 kernels generated, the parameters are selected from the following spaces: The length, *l*, is selected such that, $l \in \{7, 9, 11\}$; the value of each weight, $w_i$, in the kernel is selected such that, $w_i \sim {\mathcal {N}}(\mu ,\,\sigma ^{2})$, where $\mu = 0$ and $\sigma ^2 = 1$; dilation, *d*, is sampled from an exponential scale up to input length and the binary decision to pad the series is chosen with equal probability, if true the series is zero padded at the start and end equally such that middle element of the kernel is applied to every point in the input series. The feature spaces for parameters were learnt on a ‘development’ subset of 40 randomly selected datasets from the UCR univariate time series classification archive.

The convolution of an instance and kernel can be interpreted as the dot product between two vectors. The resulting feature map is then used to evaluate the max value and ppv features. The ppv summarises the proportion of the series correlated to the kernel. It was found to significantly improve classification accuracy. Each series is subsequently transformed into a 20,000 attribute instance after all convolutions. This transformed dataset is then used to train the ridge regression classifier.

An extension to the ROCKET approach to enable use on multivariate datasets has recently been added to the sktime repository.^2^ For multivariate datasets, kernels are randomly assigned dimensions. Weights are then generated for each channel. Convolution in this case can be interpreted as the dot product between two matrices as the kernel convolves ‘horizontally’ across the series. The max value and ppv is then calculated across all dimensions for each kernel, producing a 20,000 attribute instance.

### The multiple representation sequence learner (MrSEQL)

**Fig. 1:**
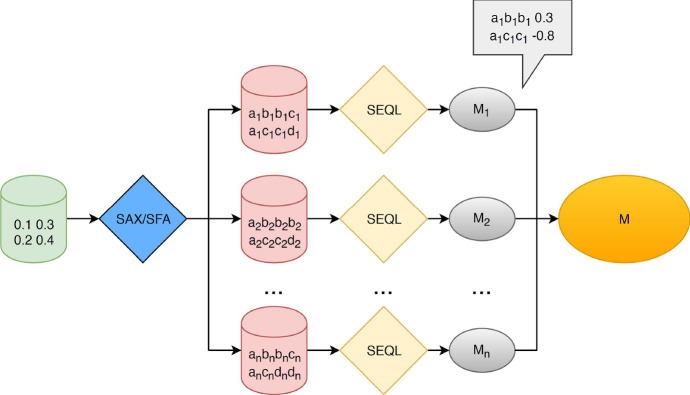
A depiction of the MrSEQL classifier taken from Nguyen et al. (2017)

The Multiple Representation Sequence Learner, MrSEQL (Le Nguyen et al. 2019) extends previous adaptations of the SEQL classifier (Nguyen et al. 2017) in two ways. Firstly, via the introduction of ensembling and secondly, via the addition of integrating the SFA (Schäfer and Högqvist 2012) transform. In the resulting approach, shown in Fig. 1, the data is transformed via either Symbolic Aggregate Approximation (SAX) (Lin et al. 2007) or SFA before being used to train a SEQL classifier. The window length, *l*, is adjusted before each addition to the ensemble. During testing each instance is transformed accordingly before being classified by the appropriate model. The output probability distribution is then the per class mean over all models.

The SEQL learner was developed for classification of biological sequences such as DNA and employs a tree based approach coupled with a pruning strategy to explore the feature space. As a result, the SFA and SAX approaches are particularly well suited as tools for transformation into the symbolic space. The SAX approach achieves this conversion by:

1. Producing a piece-wise aggregated series;
2. Creating a look-up table from the new series, in which the domain is divided by alphabet length *a*; and
3. Deriving the symbolic word, by looking up each aggregated value.

The process of aggregation and the creation of the look-up table is undertaken prior to sliding a window of length *l* across the series. At each step a word of length *w* is derived and added to the symbolic representation. The SFA approach achieves this conversion by:

1. Performing a discrete Fourier transform (DFT) on each window of the instance;
2. creating an $a \times w$ look-up table in which the alphabet boundaries are distinct for each letter index; and
3. deriving the symbolic word, by looking up each aggregated value.

The process of deriving the lookup table is undertaken after the DFT. The alphabet boundaries are then calculated per word position index. As a result there are effectively *w* alphabets of size *a*. Although not described in the original publication, the sktime version of MrSEQL classifier is implemented in such a way as it is capable of processing multivariate data. During the prepossessing of data, dimensions are processed sequentially and appended to one another creating *n* instances, each one of size $m \times c$. The implementation used in this work can be found in sktime.^3^

### Deep learning

Many of the approaches employed for MTSC are conversions of models originally designed for univariate data to handle the multivariate case. Neural networks are a natural example of this, in part due to the ease in which they can handle the extra dimensionality in the model definition and implementation.

Despite their strength and popularity in handling 2D image data, a result of AlexNet’s performance on the ImageNet dataset (Krizhevsky et al. 2012), deep learning approaches have only more recently been heavily studied in the 1D time series domain. Knowledge gained from the former can be utilised on the latter, and can now similarly be quickly transferred to the multivariate time series case. We include three deep learning approaches in our evaluation.

While (Wang et al. 2017) started with a smaller comparison of originally proposed architectures, Fawaz et al. (2019) provided the first standardised large-scale comparative study of deep learning approaches for time series classification. Nine architectures were evaluated on 85 datasets of the univariate UCR archive (Dau et al. 2019) and 13 datasets of the Baydogan multivariate archive.^4^ The Residual Network, ResNet (Wang et al. 2017) was found to be significantly better than all other approaches on the univariate datasets, and on all univariate and multivariate datasets combined. For the multivariate datasets in isolation, no significant difference was found between all approaches, mainly due the small sample size, but also due to a conservative adjustment for multiple testing. The Fully Convolutional Neural Network, FCN (Wang et al. 2017) had a slightly better overall rank, however no definitive conclusions of superiority could be drawn. We use this comparative study to take ResNet as a baseline deep learning approach for MTSC moving forward.

Currently the state-of-the-art deep learning approach for univariate time series classification is InceptionTime (Fawaz et al. 2020). To our knowledge, results for InceptionTime on multivariate archives have not been published. An approach developed specifically for multivariate timeseries classification is the Time Series Attentional Prototype Network. TapNet (Zhang et al. 2020) uses an attentional prototype network to learn the latent features.

There are currently many new deep learning architectures being proposed for time series classification. Publishing lag and difficulty in implementation or in recreating results are all reasons that methods may not appear in this comparative study. We aim for this to be a basis of easy comparison in the future rather than a final declaration of the ‘best’ algorithm. We welcome and actively encourage authors to evaluate their methods on these datasets and prove them better than those we have evaluated here.

#### Residual network (ResNet)

ResNet was first applied to time series classification in Wang et al. (2017). It is a network of three consecutive blocks, each comprised of three convolutional layers, which are connected by residual ‘shortcut’ connections that add the input of each block to its output. Residual connections allow the flow of gradient directly through the network, combating the vanishing gradient effect (He et al. 2016). The residual blocks are followed by global average pooling and softmax layers to form features and subsequent predictions. We maintain all hyperparameter settings and optimiser settings from the (Fawaz et al. 2019) evaluation, and the implementation in sktime-dl is an interfacing of the implementation provided by that study.

#### InceptionTime

InceptionTime achieves high accuracy through a combination of building on ResNet to incorporate Inception modules (Szegedy et al. 2015) and ensembling over five multiple random-initial-weight instantiations of the network for greater stability (Fawaz et al. 2020). A single network out of the ensemble is composed of two blocks of three Inception modules each, as opposed to the three blocks of three traditional convolutional layers in ResNet. These blocks maintain residual connections, and are followed by global average pooling and softmax layers as before.

**Fig. 2:**
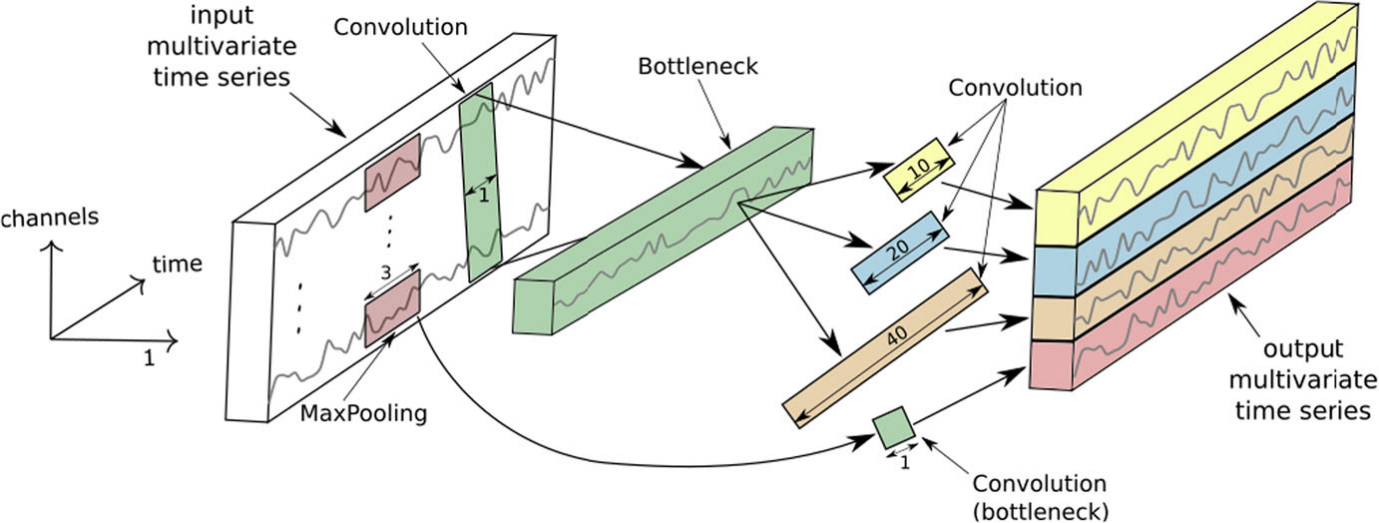
An Inception module with example parameters, figure from Fawaz et al. (2020). Three of these are concatenated to form a block in InceptionTime

An Inception module is summarised in Fig. 2. It takes an input multivariate series of length *m*, dimensionality *d*, and first uses a bottleneck layer with length and stride 1 to reduce the dimensionality to $d' < d$ while maintaining output length *m*. This greatly reduces the number of parameters to later learn. Convolutions of different lengths are applied to the output of the bottleneck layer to find patterns of different sizes. The outputs of these convolutions are combined with an additional source of diversity, a Max Pooling followed by bottleneck (with the same value of $d'$) applied to the original time series, and all stacked to form the dimensions of the output multivariate time series to be fed into the next layer.

Once more, we maintain all hyperparameter settings and optimiser settings from the source article (Fawaz et al. 2020), and the implementation in sktime-dl is an interfacing of the implementation provided by that study.

#### Time series attentional prototype network (TapNet)

A novel approach aimed at tackling problems in the multivariate domain, the TapNet architecture draws on the strengths of both traditional and deep learning approaches. Zhang et al. (2020) note that deep learning approaches excel at learning low dimensional features without the need for embedded domain knowledge whereas traditional approaches such as 1NN-DTW work well on comparatively small datasets. TapNet combines these advantages to produce a network architecture that can be broken down into three distinct modules: Random Dimension Permutation, Multivariate Time Series Encoding and Attentional Prototype Learning.

**Fig. 3:**
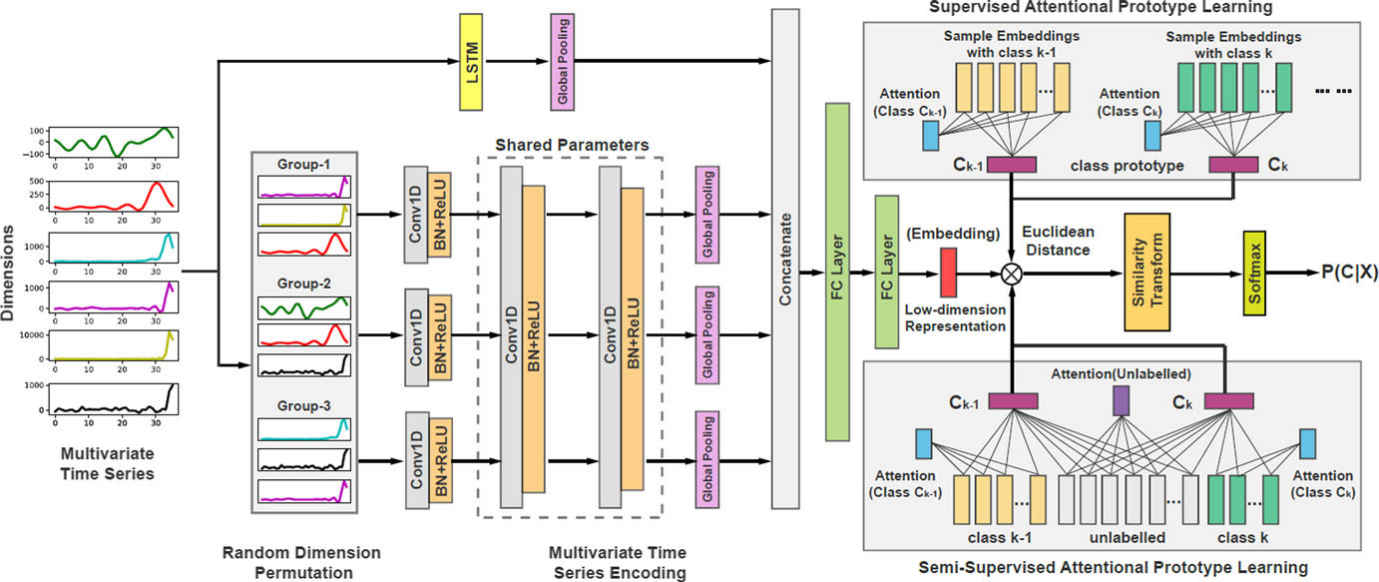
TapNet architecture, figure from Zhang et al. (2020)

Random Dimension Permutation is used to produce *g* groups of randomly selected dimensions with the intention of increasing the likelihood of learning how combinations of dimension values effect class value. The group size is defined as $\varphi = \lfloor \frac{m \cdot \alpha }{g} \rfloor$, where $\alpha$ is the scale factor, controlling the number of dimensions used over *m*, where *m* is the number of dimensions. This process is illustrated in Fig. 3 where the six input dimensions are reorganised into three groups of three. Experimentation exploring the effect of this module found that in 22 out of 33 datasets in the UEA multivariate archive the accuracy was increased. However, it is unclear whether it has a significant effect or whether the effect on accuracy is a function of dataset characteristics.

Encoding in the TapNet architecture is undertaken in $g + 1$ stages before the output features are concatenated and passed through two fully connected layers. Each group produced in the dimension permutation module is passed through three sets of one-dimensional convolutional layers followed by batch normalisation, Leaky Rectified Linear Units and finally a global pooling layer. For the first of these three sets the weights and bias are distinct for each group. In addition to the group encoding process, the raw data is passed through an LSTM and global pooling layer. The output from each of the global pooling layers are then concatenated before being passed through two fully connected layers. This process results in a low-dimensional feature representation of the original series. The default filter values for the convolution layers are set as 256, 256 and 128 whilst the default kernel values are five, eight and three. The default value for the LSTM layer is 128. It is intended that interaction between dimensions can be learned more effectively by the Random Dimension Permutation process before the encoding is then combined, producing features aligned with a datasets dimensions. Furthermore, the inclusion of the LSTM layer is intended to learn longitudinal features.

Finally, for each class a prototype candidate is produced. Although the architecture does allow for unlabelled test data to be included in the prototype derivation via Semi-supervised Attentional Prototype Learning. This feature was not utilised. As a result, the class prototypes are defined solely by the training data. The objective of the candidate production is to minimise the distance to all members of the class which the prototype is produced for whilst maximising the distance between the prototypes. Probability of class membership is then assigned to test instances as a function of their proximity to each class prototype. In this case the similarity is measured by way of Euclidean distance.

## The UEA multivariate time series classification archive

Research into MTSC is in a position where univariate TSC research was a decade ago. Algorithms are evaluated using very few datasets and claims of improvement are not based on statistical comparisons. Recent research has improved somewhat because of the assembly of an archive of datasets by Mustafa Baydogan.^5^ This archive is useful, and appears many times in the literature e.g. Fawaz et al. (2019), Schäfer and Leser (2018), Karlsson et al. (2016), Baydogan and Runger (2016), but it has limitations. The data are generally small, are not independent, are mostly variable length and are not representative of many important MTSC domains. The UEA MTSC archive (Bagnall et al. 2018) was formed to overcome these problems. On release in 2018 it contained 30 multivariate datasets, of which four are not all equal length. To focus on classification rather than preprocessing issues, we restrict our attention to the 26 equal length series. The main characteristics of each problem are summarised in Table 1. Details can be found on the associated website.^6^

**Table 1:** Summary of the 26 UEA datasets used in experimentation

Code	Name	Train size	Test size	Dims	Length	Classes
AWR	ArticularyWordRecognition	275	300	9	144	25
AF	AtrialFibrillation	15	15	2	640	3
BM	BasicMotions	40	40	6	100	4
CR	Cricket	108	72	6	1197	12
DDG	DuckDuckGeese	50	50	1345	270	5
EW	EigenWorms	128	131	6	17,984	5
EP	Epilepsy	137	138	3	206	4
EC	EthanolConcentration	261	263	3	1751	4
ER	ERing	30	270	4	65	6
FD	FaceDetection	5890	3524	144	62	2
FM	FingerMovements	316	100	28	50	2
HMD	HandMovementDirection	160	74	10	400	4
HW	Handwriting	150	850	3	152	26
HB	Heartbeat	204	205	61	405	2
LIB	Libras	180	180	2	45	15
LSST	LSST	2459	2466	6	36	14
MI	MotorImagery	278	100	64	3000	2
NATO	NATOPS	180	180	24	51	6
PD	PenDigits	7494	3498	2	8	10
PEMS	PEMS-SF	267	173	963	144	7
PS	PhonemeSpectra	3315	3353	11	217	39
RS	RacketSports	151	152	6	30	4
SRS1	SelfRegulationSCP1	268	293	6	896	2
SRS2	SelfRegulationSCP2	200	180	7	1152	2
SWJ	StandWalkJump	12	15	4	2500	3
UW	UWaveGestureLibrary	120	320	3	315	8

### Electrical biosignals

Electrocardiograms (ECG), Electroencephalograms (EEG) and Magnetoencephalography (MEG) are all techniques for measuring, directly or indirectly, actual or relative changes in voltage throughout the body. They are also all inherently multivariate as typically several readings are produced and used for interpretation. ECGs are typically used to detect and measure the electrical activity of the heart. EEGs are used to measure brain activity (brain waves), and are typically used in the diagnosis of epilepsy and seizures. Both ECGs and EEGs measure voltage or the potential difference between points directly. However, MEGs are designed to record the magnitude of the magnetic field produced by the brain. As a result they can produce data with a high temporal and spatial resolution. Many applications associated with electrical biosignal datasets revolve around human/computer interfacing or autonomous anomaly detection.

#### AtrialFibrilation (Goldberger et al. 2000)

This dataset of two-channel ECG recordings has been created from data used in the Computers in Cardiology Challenge 2004, an open competition with the goal of developing automated methods for predicting spontaneous termination of atrial fibrillation (AF). The raw instances were 5 s segments of atrial fibrillation, containing two ECG signals, each sampled at 128 samples per second. The multivariate data organises these dimensions such that each is one dimension. The class labels are: n, s and t. Class n is described as a non termination atrial fibrillation (that is, it did not terminate for at least 1 h after the original recording of the data). Class s is an atrial fibrillation that self terminates at least 1 min after the recording process. Class t is described as terminating immediately, that is within 1 s of the recording ending.

#### FaceDetection

This dataset consists of MEG recordings.^7^ Whilst recording data participants were shown either a scrambled picture or one showing a face. The raw data consists of 306 dimensions of 375 attributes. We use data with a reduced set of 144 dimensions which is also provided on the competition website. The training set is comprised of recordings from 10 individuals, whilst the test set is comprised of a separate 6 individuals. Each participant contributed between 580 and 590 instances.

#### FingerMovements (Blankertz et al. 2002)

This dataset consists of 500 ms intervals of EEG recordings 130 ms prior to the moment a key is pressed. A single subject, sat in a normal position at a keyboard was asked to type characters using only the index and pinky fingers. The dataset consists of 28 dimensions of 50 attributes. The training set contains 316 instances while the test set contains 100. There are two target classes: left and right.

#### HandMovementDirection

In this dataset two right handed subjects were recorded moving a joystick with their hand and wrist only in one of four directions (right, up, down, left) of their choice after hearing a prompt.^8^ Using the resulting MEG, the task is to classify the direction of movement. Each recording represents an interval starting 0.4 s before the movement and ending 0.6 s afterwards. Instances have a sample rate of 400 Hz.

#### MotorImagery (Lal et al. 2005)

This dataset consists of 64 dimension EEG data. The data was generated by a participants imagined movement of either their little finger or tongue. An eight by eight Electrocorticography platinum grid was placed over the right motor cortex during the data generating process. Recordings were initiated 0.5 s after a visual cue had ended and are three 3 s in duration. The train and test sets were recorded in the exact same fashion. However, they were recorded 1 week apart.

#### SelfRegulationSCP1 (Birbaumer et al. 1999)

Healthy participants were asked to visualise moving a cursor either up or down on a screen. The direction of travel was determined via their Slow Cortical Potential, measured via EEG and fed back to the participant visually. The EEG data was taken from 6 positions on the head. The object of this problem is to classify each instance as positive (downward) and negative (upward) movement, based form the EEG readings.

#### SelfRegulationSCP2 (Birbaumer et al. 1999)

An artificially respirated ALS patient was asked to move a cursor either up or down on a screen. The direction of travel was determined via their Slow Cortical Potential, measured via EEG and fed back to the participant visually and audibly. The EEG data was taken from seven positions on the head. The object of this problem is to classify each instance as positive (downward) or negative (upward) movement, based form the EEG readings.

#### StandWalkJump (Goldberger et al. 2000)

Short duration ECG signals were recorded from a healthy 25-year-old male performing different physical activities to study the effect of motion artifacts on ECG signals and their sparsity. The raw data was sampled at 500 Hz, with a resolution of 16 bits before an analogue gain of 100 and ADC was applied. A Spectrogram of each instance was then created with a window size of 0.061 s and an overlap of 70%. Each instance in this multivariate dataset is arranged such that each dimension is a frequency band from the spectrogram. There are three classes: standing, walking and jumping, each consists of nine instances.

### Accelerometer/gyroscope

An accelerometer measures change in speed and typically devices are capable of reporting information on all three axis of movement (*x*, *y*, *z*). They are useful in measuring events such as impacts or vibration. Many datasets are used to investigate whether the various vectors of acceleration produced during a variety of tasks produces enough discriminatory information for classification. Gyroscopes measure angular velocity and provide an indication to the extent a device has rotated about each axis.

#### BasicMotions

This dataset was collected by students at UEA. Data was generated by participants performing four activities whilst wearing a smart watch. The watch collects 3D accelerometer and gyroscope data. The dataset consists of four classes: walking, resting, running and badminton. Participants were required to record each motion a total of five times. The sample rate of both sensors was 10 Hz and activity was recorded for 10 s.

#### Cricket (Ko et al. 2005)

Cricket requires an umpire to signal different events in the game to a distant scorer. The signals are communicated with motions of the hands. For example, No-Ball is signalled by touching each shoulder with the opposite hand, and TV-Replay (a request for an off-field review of the video of a play) is signalled by miming the outline of a TV screen.

This dataset consists of four umpires performing 12 signals, each with ten repetitions. The data, recorded at a frequency of 184 Hz, was collected by placing accelerometers on the wrists of the umpires. Each accelerometer has three synchronous measures for three axes (x, y and z). Thus, we have a six-dimensional problem from the two accelerometers.

#### Epilepsy (Villar et al. 2016)

Data was collected from six participants using a 3D accelerometer on the dominant wrist. All examples of the four classses: walking, running, sawing and seizure mimicking (whilst seated), were recorded for different lengths of time. The sampling frequency was 16 Hz. Each participant performs each activity ten times at least. The mimicked seizures were trained and controlled, following a protocol defined by a medical expert.

Some activities lasted about 30 s, others are 1 min long, others are about 2 min. This data was truncated to the length of the shortest series retained prior to our policy of retaining data as unequal length problems. We removed prefix and suffix flat series and truncated to the shortest series (20 measurements, approx 13 s), taking a random interval of activity for series longer than the minimum. A single case from the original (ID002 Running 16) was removed because the data was not collected correctly. After tidying the data we have a total of 275 cases. The train test split is divided into three participants for training, three for testing, with the IDs removed for consistency with the rest of the archive.

#### Handwriting (Shokoohi-Yekta et al. 2017)

Accelerometer data recorded whilst a subject writes all 26 letters of the alphabet. The watch was worn on the same wrist as used to write. The dataset consists of 150 train cases and 850 test cases.

#### NATOPS (Ghouaiel et al. 2017)

Adapted from the 2016 Advanced Analytics and Learning on Temporal Data challenge.^9^ This 24 dimension data was recorded via Xbox Kinect whilst participants performed one of six gestures. Sensors attached to each: hand, elbow, wrist and thumb recorded the position in 3D space throughout the gesture.

#### RacketSports

This dataset was collected by students at UEA. The dataset consists of data captured whilst participants played one of two strokes whilst playing badminton or squash. The data was captured via a smart watch (Sony Smart watch 35), worn on the dominant hand. The watch relayed the x, y, z values for both the gyroscope and accelerometer at a rate of 10 HZ over 3 s whilst the player played either a forehand/backhand in squash or a clear/smash in badminton.

#### UWaveGestureLibrary (Liu et al. 2009)

This dataset consists of 3D accelerometer data captured during the performance of a gesture. There are 8 gestures (classes) and 440 instances in total, each series is 315 long.

### Coordinates

Typically recorded in Cartesian space, in these problems an objects location is tracked, either relative to a start position or in the context of some larger environment. Many of these examples revolve around gesture and digit recognition, but the data is distinct to accelerometer/gyroscope data, since coordinates may be extracted from images or bespoke hardware.

#### ArticularyWordRecognition (Wang et al. 2013)

An Electromagnetic Articulograph (EMA) is an apparatus used to measure the movement of the tongue and lips during speech. The motion tracking using EMA is registered by attaching small sensors on the surface of the articulators (e.g., tongue and lips). Subjects are then seated within a calibrated magnetic field. As a result the changes in sensor position can be measured. The spatial accuracy of motion tracking using EMA AG500 is 0.5 mm. Data was collected from multiple native English native speakers producing 25 words. Nine sensors were used in data collection, each providing *x*, *y* and *z* positions with a sampling rate of 200 Hz. Four sensors were located along the mid-line of the tongue, one sensor was located in the centre of the top lip and another was located in the centre of the bottom lip. Of the total of 27 available dimensions, this data set includes just nine.

#### LIBRAS (Dias and Peres 2016)

This dataset contains 15 classes each made up of 24 instances each. Each class references a hand movement from the Brazilian sign language, LIBRAS. Each instance represents a gesture extracted from a video and transformed to 2D coordinate space. The videos used contained four different subjects. Each 7 s video contained 1 hand movement. From each video 45 frames were selected uniformly to extract the hand positions from.

#### PenDigits (Alimoğlu and Alpaydin 2001)

This dataset contains bi-dimensional (x, y) coordinate data regarding pen location during a writing task. 44 participants were asked to write the digits 0–9. The data is normalised and from expert knowledge the data was spatially resampled such that each consecutive attribute has a constant spatial step and variable time step. From experimentation by the authors, the data was resampled to 8 spatial points, such that each instance is 2 dimensions of 8 points.

### Audio

Audio is a quintessential example of time series data and at the heart of many real world machine learning applications. Typically, we interact with audio data in its univariate time domain form. Furthermore, it is commonly accepted that features extracted from the spectral domain provide more predictive power than those from the time domain. However, spectral features in this format are time agnostic and as a result approaches are unable to leverage information on how the power of spectral coefficients changes over time. For the purpose of this archive we present audio problems in a spectrogram format. This format exposes the spectral decomposition of the data and expresses the change in spectral power over time. This presents an opportunity to evaluate novel approaches that can leverage this extra dimension.

#### DuckDuckGeese

This dataset was derived from recordings found on the Xeno Canto website.^10^ Each recording was taken from either the A or B quality category. Due to the variation in recorded sample rate all recordings were downsampled to 44,100 Hz using the MATLAB resample function. Each recording was then center truncated to 5 s (length of smallest recording), before being transformed into a spectogram using a window size of 0.061 and an overlap value of 70%. The classes are as follows: Black-bellied Whistling Duck (20 instances); Canadian Goose (20 instances); Greylag Goose (20 instances); Pink Footed Goose (20 instances); and White-faced Whistling Duck (20 instances).

#### Heartbeat (Goldberger et al. 2000)

This dataset is derived from the PhysioNet/CinC Challenge 2016.^11^ Heart sound recordings were sourced from both healthy subjects and pathological patients and recorded in a clinical environment. The heart sound recordings were typically collected from one of four locations. The sounds were divided into two classes: normal and abnormal. The normal recordings were from healthy subjects and the abnormal ones were from patients with a confirmed cardiac diagnosis. Each recording was truncated to 5 s. A Spectrogram of each instance was then created with a window size of 0.061 s and an overlap of 70%. Each instance in this multivariate dataset is arranged such that each dimension is a frequency band from the spectrogram. The two classes normal and abnormal consist of 113 and 296 instances respectively.

#### Phoneme (Hamooni and Mueen 2014)

This dataset is a multivariate representation of a subset of the data used in Hamooni and Mueen (2014). Each series was extracted from the segmented audio collected from Google Translate. Audio files collected from Google translate are recorded at 22,050 HZ. The speakers are male and female. After data collection, they segment waveforms of the words to generate phonemes using the Forced Aligner tool from the Penn Phonetics Laboratory. A Spectrogram of each instance was then created with a window size of 0.001 s and an overlap of 90%. Each instance in this multivariate dataset is arranged such that each dimension is a frequency band from the spectrogram. The data consists of 39 classes each with 170 instances.

### Other datasets

In the interest of brevity datasets produced using a technique novel to the archive are collected here. These include datasets produced via spectrometry, photometry and bespoke hardware. We hope that these domains will increase in size and encourage users to either submit or suggest new sources of data.

#### ERing (Wilhelm et al. 2015)

This data is generated with a prototype finger ring, called eRing, that can be used to detect hand and finger gestures. eRing uses electric field sensing rather than motion. This data set is the D data set used for Finger Posture Recognition. There are six classes for six postures involving the thumb, the index finger, and the middle finger. The data is four dimensional. Each series contains 65 observations. Each series is a measurement from an electrode which varies dependent on the distance to the hand.

#### EthanolConcentration (Large et al. 2018)

EthanolConcentration is a dataset of raw spectra taken of water-and-ethanol solutions in 44 distinct, real whisky bottles. The concentrations of ethanol are 35%, 38%, 40%, and 45%. The minimum legal alcohol limit for Scotch Whisky is 40%. Producers are required to ensure that the contents of their spirits contain alcohol concentrations that are tightly bound to what is reported on the labelling. The classification problem is to determine the alcohol concentration of a sample contained within an arbitrary bottle. In this formulation, there are four classes, corresponding to the four concentrations.

The data has been arranged such that each instance is made up of three repeat readings of the same bottle and batch of solution. Three solutions of each concentration (batches) were produced, and each bottle+batch combination measured three times. Each reading is comprised of the bottle being picked up, placed between the light source and spectroscope, and spectra saved. The spectra are recorded over the maximum wavelength range of the single StellarNet BLACKComet-SR spectrometer used (226–1101.5 nm with a sampling frequency of 0.5 nm), over a 1 s integration time. Except for avoiding labelling, embossing, and seams on the bottle, no special attempts were made to obtain the cleanest reading for each individual bottle, nor to precisely replicate the exact path through the bottle for each repeat reading. This is to replicate potential field-conditions in the future of an operative performing mass-screening of a batch of suspect spirits.

#### LSST

The LSST dataset was adapted from the Photometric LSST Astronomical Time Series Classification Challenge (PLAsTiCC).^12^ It consists of astronomical time series data. Each time series is a ‘light curve’ and measures an object’s brightness as a function of time. By measuring the photon flux in six different astronomical filters (commonly referred to as passbands) the objective is to classify the class of astronomical object.

#### PEMS-SF (Cuturi 2011)

Made available by the Department of Transportation,^13^ this dataset represents 15 months worth of traffic data from various locations on the San Francisco bay area freeway network. Data was recorded from each of 963 stations (dimensions) every 10 min. Each instance is 144 attributes long and equates to 1 day. The objective is to classify which day of the week each instance was recorded on.

## Methods

Our experiments are designed to assess the relative merits of the algorithms in terms of performance and usability over a range of datasets. We take DTW as our benchmark algorithm. In Sect. 4.1 we describe the algorithm implementations we have used. All algorithms have been implemented either by, or in consultation with, the person or group who originally proposed the method. The nature of the experiments and performance metrics used are outlined in Sect. 4.3.

### Toolkits

One barrier to reproducible research is the incompatibility of software used to generate results across different projects. To overcome this problem, we help maintain two toolkits that include time series classification functionality. sktime^14^ is an open source, Python based, sklearn compatible toolkit for time series analysis. sktime is designed to provide a unifying API for a range of time series tasks such as annotation, prediction and forecasting. See Löning et al. (2019) for a description of the overarching design of sktime and Bagnall et al. (2019) for an experimental comparison of some of the classification algorithms available. The Java toolkit for time series machine learning, tsml,^15^ is Weka compatible and is the descendent of the codebase used to perform univariate TSC benchmarking (Bagnall et al. 2017). The two toolkits will eventually converge to include all classifiers described. To reduce the number of dependencies in the core package, sktime has subpackages for specific forms of classification. sktime-dl provides a range of deep learning approaches to time series classification and sktime-shapelets-forest gives shapelet functionality.^16^ The mechanism for running an experiment for a combination of classifier, problem and resample (‘single evaluation’, henceforth) are the same in both toolkits. Available classifiers are given in ClassifierLists.java and classifier_lists.py. Usage with tsml Experiments.java is shown in code listing 1. The equivalent with sktime class experiments.py is shown in listing 2.
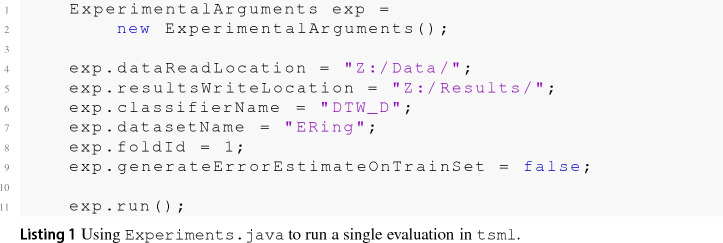

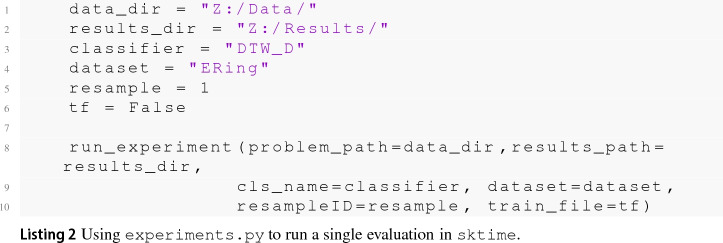


The format of the results for a single evaluation is the same for both toolkits. Iterating over all classifier/problem/resample combinations will generate a test results file containing test predictions and results for each single evaluation. If generateTrainFiles is set to true, an external cross validation (or internal performance estimation mechanism if available) on the train data for that resample will be used to generate a train results file. The dimension independent ensembles can be built in both toolkits using a dimension ensemble. In tsml this is can be done with, for example, the RISE classifier as follows:
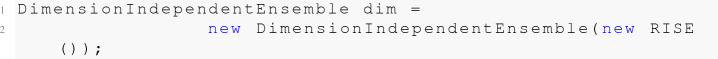


In sktime it is called a ColumnEnsembleClassifier, and can be configured thus: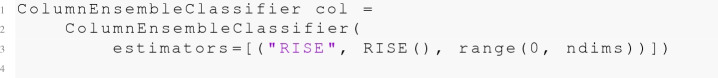 It is possible to build HIVE-COTE from DimensionIndependentEnsemble elements, but computational resources can likely be better utilised if each component is built independently (with generate train files set to true) and then ensembled with HIVE-COTE later from the results files.
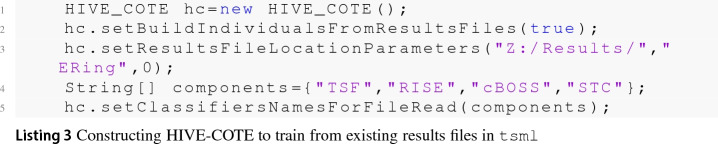
 Table 2 lists the algorithms and their availability in the toolkits. Where a classifier is available in both toolkits, we run experiments in tsml, because it is generally faster. TapNet is the only algorithm not yet ported to a toolkit. We are working to include it in sktime-dl.

**Table 2:** Classifier availability in the two toolkits tsml and sktime

Algorithm	tsml	sktime	sktime-dl	sktime-shapelets
DTW_D	X	X		
DTW_I	X	X		
DTW_A	X			
MUSE	X			
gRFS				X
MrSEQL		X		
ROCKET		X		
CIF	X	X		
TapNet				
ResNet			X	
InceptionTime			X	
CBOSS	X	X		
STC	X	X		X
RISE	X	X		
TSF	X	X		
HIVE-COTE	X	X		

### Evaluation and comparison of classifiers

For every classifier, we average performance measures over the thirty resamples to present a single statistic for each dataset/classifier combination. We analyse the results files using evaluation code in tsml. This code collates all the results, summarises a large range of performance metrics (accuracy, AUC, F1 etc), conducts statistical tests to compare classifiers and draws comparative diagrams such as scatter plots and critical difference diagrams. The results collated by MultipleClassifierEvaluation (MCE) (see Listing 4) include performance metrics (accuracy, area under the ROC, balanced accuracy, F1, negative log likelihood, Matthew’s correlation coefficient, recall/sensitivity, precision and specificity). When stored in the problem files, it also collates memory usage and run time. By default, MCE compares pairs of classifiers using the Wilcoxon sign rank test, and presents the relative results as scatter plots and critical difference diagrams generated in Matlab. These graphs are explained in more detail when first used. In Sect. 5 we present a selection of the results generated by MCE for brevity. However, all these results are available on the associated website. Our main focus is on accuracy due to its ease of motivation and interpretation on arbitrary datasets, but we also present the area under the receiver operator curve (AUROC), balanced accuracy and F1 statistics.
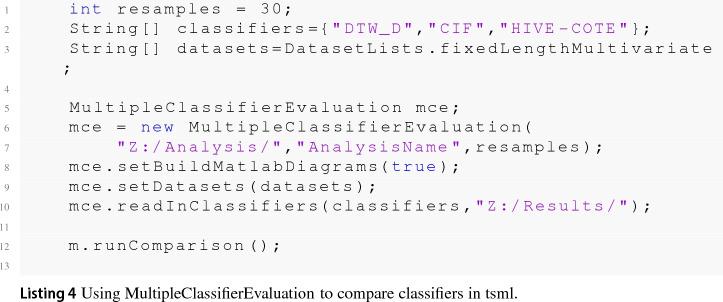
 For pairwise comparison of two classifiers, by default we follow the standard machine learning approach of using the non parametric Wilcoxon sign rank test. For some tests, we have also performed a paired *t*-test for contrast. To compare multiple classifiers on multiple data sets, we adapt the approach from Demšar (2006) and use critical difference diagrams. These order classifiers by rank, and group classifiers together into cliques, sets of classifiers between which there is no significant difference. Based on the literature (Benavoli et al. 2016), we abandon the post hoc test used in Demšar (2006) and instead form cliques with pairwise tests, making the Holm correction for multiple testing. For the majority of diagrams we use the Wilcoxon sign rank test for pairwise comparison. However, for completeness and as a basic sanity test, we also show the results of paired *t*-tests for the most important results.

### Experiments

Experiments with tsml, sktime and sktime-shapelets-forest were conducted on the UEA high performance computing (HPC) cluster. The nature of the HPC means that any one job (a single evaluation) runs on a single core and has a maximum execution time of 7 days. For memory intensive algorithms, we reran with increasing memory until successful completion, up to a maximum of 500 GB.

Experiments with sktime-dl and TapNet were performed on GPUs in desktops, one with a Titan XP and one with four Titan X Pascals. All jobs were run on a single GPU, and each GPU ran only one job at a time. There was no time limit for these jobs. However, the jobs were limited by the GPU memory of 12 GB per card.

For each dataset, we perform thirty stratified resamples (maintaining the class distribution in the original train/test splits) and store all test predictions. The first resample is always the original train/test split. The remaining splits are seeded by the resample number and are reproducible. Therefore, all classifiers are given identical resamples for all problems.

Table 3 summarises the set configurations used. All of these are the default settings specified by the authors. Further details are available on the associated web page. Some classifiers perform internal tuning as part of their original algorithm definition, but we have done no external tuning unless it was explicitly preformed in the paper proposing the algorithm and the code provided makes it possible to do so.

**Table 3:** Classifier configuration

Algorithm	Configuration
DTW_D	Full warping window
DTW_I	Full warping window
DTW_A	Full warping window
MUSE	$\chi =2$
gRFS	Default (max depth: none, min sample split: 2, num shapelets: 10
	Min size: 0%, max size: 100%, metric: Euclidean distance)
MrSEQL	seql_mode: fs, symrep: [’sax’, ’sfa’]
ROCKET	Ridge regression classifier, 10,000 kernels
CIF	Default (trees: 500, intervals: $\sqrt{(}m) \times \sqrt{(}d)$, 8 attributes per tree)
TapNet	Default (Epochs: 3000, Learning rate: $1e{-}5$, weight decay: $1e{-}3$
	Stop threshold: $1e{-}9$, num filters: [256 256 128], kernels: [8 5 3]
	Dilation: 1, dropout: 0%)
	ArticularyWordRecognition (dilation: 10)
	EthanolConcentration (dilation: 200, learning rate: $1e{-}6$)
	FaceDetection (filters: [64 64 32], learning rate: $5e{-}5$)
	Heartbeat (dilation: 200, learning rate: $1e{-}6$, filters: [64 64 32])
	PenDigits (kernels: [4 1 1], learning rate: $1e{-}3$)
	PhonemeSpectra (filters: [64 64 32], learning rate: $1e{-}3$)
	SelfRegulationsCP1 (learning rate: $1e{-}6$)
	SelfRegulationsCP2 (learning rate: $1e{-}9$)
	SpokenArabicDigits (filters: [128 128 64], learning rate: $1e{-}4$)
ResNet	Epochs: 1500, batch size: 16, learning rate: $1e{-}3$ and halved after
	No improvement for 50 epochs
	Three residual blocks each with three conv layers with kernel sizes [8, 5, 3]
	Filters per conv layer for each block [64, 128, 128]
InceptionTime	Epochs: 1500, batch size: 64, learning rate: $1e-3$ and halved after no
	Improvement for 50 epochs
	Two residual blocks each with three Inception modules with kernel sizes
	Per module [10, 20, 40]
	Plus bottleneck filters for all conv layers 32
CBOSS	Default, see Bagnall et al. (2020)
STC	Default, see Bagnall et al. (2020)
RISE	Default, see Bagnall et al. (2020)
TSF	Default, see Bagnall et al. (2020)
HIVE-COTE	Version 1.0 Bagnall et al. (2020)

**Fig. 4:**
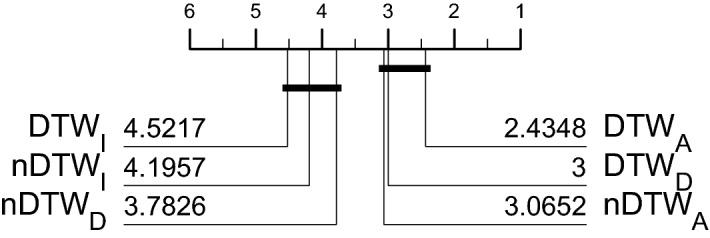
Critical difference diagram for three versions of DTW, with the data normalised (prefix n) or not normalised (no prefix). EigenWorms is not included in these results since $\hbox {DTW}_A$ did not complete the problem

**Fig. 5:**
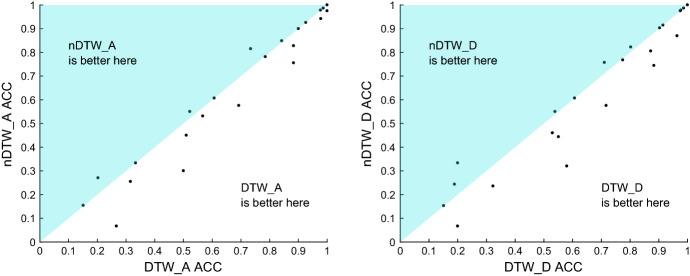
Scatter plots of accuracy on 26 UEA MTSC problems for $\hbox {DTW}_A$ and $\hbox {DTW}_D$ built with normalised (labelled $\hbox {nDTW}_A$ and $\hbox {nDTW}_D$) and non normalised data. Normalisation was performed independently on each dimension so that every series has zero mean and unit standard deviation

Another question to resolve is whether to normalise the data or not. The majority of past research has assumed it is always best to normalise the time series. The reasoning for this is two fold: firstly, it is claimed that if summary measures such as mean and variance can be used to discriminate, then the problem is trivial. Secondly, not normalising a series can distort comparisons of algorithms, some of which internally normalise the data. We have sympathy with both arguments. However, particularly with multivariate data, we do not think it so simple. Scale and variance in one dimension may be discriminatory factors without trivialising the problem. This is particularly relevant to MTSC, where interactions in shape, level and variance may be needed to find the best classifier. We think the best approach is to present the data to the classifiers with no preprocessing, so we do not normalise all data prior to building a classifier. ROCKET, gRSF, TapNet, InceptionTime, ResNet, CBOSS, STC and CIF all use some form of internal normalisation, whereas MUSE treats normalisation as a parameter. Hence, the danger of unfair comparison is real, particularly with the DTW algorithms. To assess this problem, we have run the three DTW variants on normalised and unnormalised data. Normalisation is performed independently on each dimension, so that the series have zero mean and unit variance. Figure 4 shows the critical difference diagram for the DTW versions built using both normalised and unnormalised data on the default train/test split. The number by each classifier indicates its average rank (lower is better) and the solid lines indicate groups of classifiers within which there is no significant difference in rank. These are formed by testing the highest ranked vs the next highest (using a correction for multiple testing) until a difference is found. The process is then repeated until the lowest rank classifier is either in a clique or on its own. There are two cliques in these results: ($\hbox {DTW}_A$, $\hbox {DTW}_D$, $\hbox {nDTW}_A$) and ($\hbox {nDTW}_D$, $\hbox {nDTW}_I$, $\hbox {DTW}_I$). Forming cliques with pairwise tests is the best procedure (Benavoli et al. 2016), but it can be deceptive when the classifiers are very similar. A clique contains classifiers with no pairwise difference between them. However, that does not mean there is always a significant difference between classifiers in different cliques. Table 4 shows the results of the pairwise tests used to form Fig. 4, with the two cliques in bold. It shows that, although in different cliques, there is, for example, no significant difference between $\hbox {nDTW}_A$ and $\hbox {DTW}_I$. Generally, normalisation makes DTW worse, but it is always worth visualising the differences. Figure 5 shows the performance of $\hbox {DTW}_A$ with and without normalisation. All three were worse after normalisation on average, $\hbox {DTW}_D$ and $\hbox {DTW}_A$ significantly so. We also built HIVE-COTE and its components on normalised series, and found no significant difference. We do not conclude that normalisation is unnecessary, merely that, for these experiments, not normalising is not going to bias against the baseline DTW classifiers.

**Table 4:** Results of pairwise tests of significance for the normalised and not normalised DTW experiments

	$\hbox {DTW}_A$	$\hbox {DTW}_D$	$\hbox {nDTW}_A$	$\hbox {nDTW}_D$	$\hbox {nDTW}_I$	$\hbox {DTW}_I$
$\hbox {DTW}_A$	**True**	**True**	**True**	False	False	False
$\hbox {DTW}_D$	**True**	**True**	**True**	True	False	False
$\hbox {nDTW}_A$	**True**	**True**	**True**	False	True	True
$\hbox {nDTW}_D$	False	True	False	**True**	**True**	**True**
$\hbox {nDTW}_I$	False	False	True	**True**	**True**	**True**
$\hbox {DTW}_I$	False	False	True	**True**	**True**	**True**

A cell is labelled true if there is no significant difference using a paired Wilcoxon sign rank test, $\alpha =0.05$

**Fig. 6:**
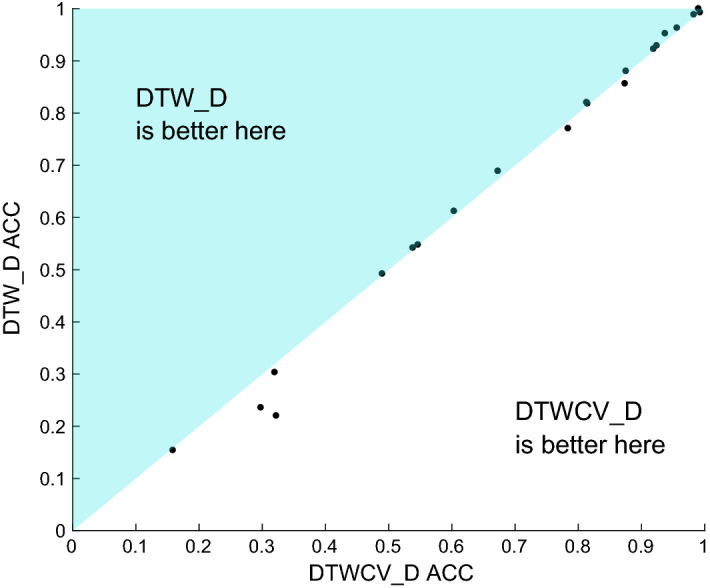
Scatter plot for $\hbox {DTW}_D$ and $\hbox {DTWCV}_D$ on 21 of the 26 UEA MTSC problems

Finally, we need to determine whether to tune the DTW window size. For the univariate archive, tuning the window parameter gives a small, but significant, overall improvement over using the full window size Ratanamahatana and Keogh (2005). To use DTW as a baseline, we need to assess whether this improvement is also observable for MTSC. Tuning is time consuming, and in order to complete the large problems we would need to include numerous known speed improvements (Tan et al. 2018) which also come with a huge memory overhead. We ran a naive implementation $\hbox {DTW}_D$ with all window sizes from 0 to 100% evaluated with cross validation. This completed on 21 of 26 problems within our time limit. Figure 6 shows the scatter plot of $\hbox {DTW}_D$ vs the tuned version $\hbox {DTWCV}_D$ on the unnormalised data. Untuned $\hbox {DTW}_D$ is better on 14 of the 21, but overall there is no significant difference and no observable benefit from tuning. As with normalisation, we are not claiming that tuning is not worthwhile generally. We do claim that for the purposes of this study, there is no reason to tune the baseline algorithm DTW, since it makes no practical difference in terms of classification accuracy. Based on these results, we conclude that $\hbox {DTW}_D$ should be the benchmark for comparison rather than $\hbox {DTW}_I$, and that to do so without normalisation or tuning is acceptable in this context.

## Results

We could not obtain results for all algorithms on all datasets within our constraints. $\hbox {DTW}_A$ did not complete Eigenworms within the 7 day limit, and InceptionTime could not complete Eigenworms due to out-of-memory errors on the GPU. MrSEQL failed to finish FacedDetection and PhonemeSpectra in time. TapNet completed on 23 datasets, but could not allocate enough memory for PhonemeSpectra, EigenWorms and MotorImagery. The bottleneck for MUSE is memory. It failed to complete six problems: DuckDuckGeese; EigenWorms; FaceDetection; MotorImagery; PEMS-SF; and PhonemeSpectra. We ran gRSF with default parameters on all datasets without problems. However, tuning with the recommended parameter ranges (Karlsson et al. 2016) proved infeasible. Only nine of the 26 experiments completed in 7 days.

It is possible we could have engineered these algorithms and their parameter spaces to work on the problematic datasets. However, our goal is to test classifiers based on the configuration recommended by the original authors. We do not want to bias our results by optimising algorithms for particular datasets. All 16 classifiers completed 20 problems, 11 classifiers completed all 26 problems. We could have given the algorithms more than 7 days to to run for the missing problems. However, none of these problems are truly large by modern data standards (the biggest train file is 500 MB), and a 7 day run with no external tuning seems a reasonable limit.

Table 11 in “Appendix 1” presents the accuracy results of all 16 classifiers on all problems. Each data is the average statistic over 30 resamples. The default train/test results are provided on the associated website. We split the detailed analysis into two parts: a comparison of algorithms that complete all 26 problems (Sect. 5.1) and a comparison of all classifiers on the reduced set of 20 problems (Sect. 5.2). We then explore performance by dataset to assess the usefulness of the archive in Sect. 5.3.

**Fig. 7:**
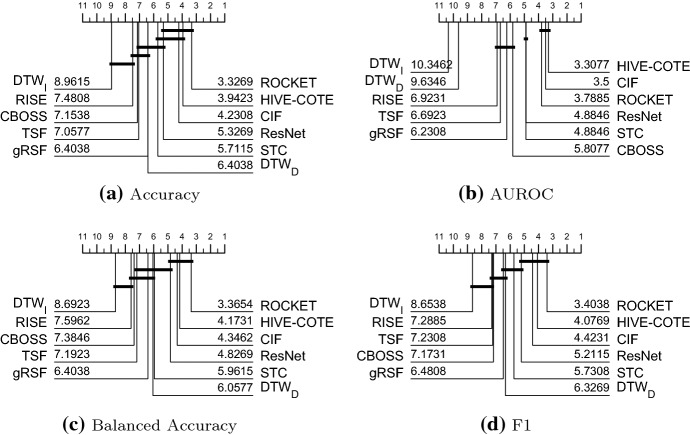
Critical difference diagrams for 11 classifiers on the 26 equal length UEA datasets using pairwise Wilcoxon test to form cliques

### Comparison of eleven classifiers on twenty six datasets

**Fig. 8:**
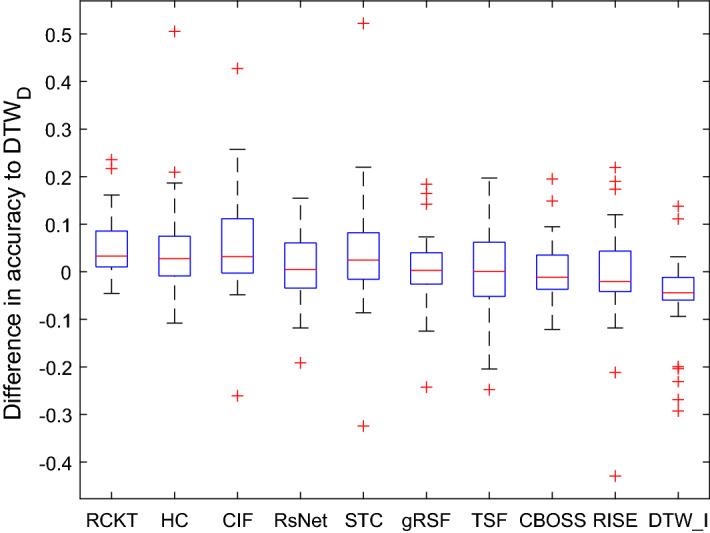
Box plots of the differences in accuracy relative to $\hbox {DTW}_D$ over datasets

Figure 7 shows the critical difference diagrams for the 11 classifiers that completed all 26 problems, with cliques formed with the rank based Wilcoxon tests. The top clique is (ROCKET, HIVE-COTE, CIF, ResNet) and the top three classifiers are all significantly more accurate than the baseline $\hbox {DTW}_D$. The middle cliques indicate that there is no significant difference between $\hbox {DTW}_D$ and any of the other classifiers except $\hbox {DTW}_I$, which is significantly worse. Balanced accuracy and F1 give a very similar pattern of results, indicating class imbalance is not a factor. $\hbox {DTW}_D$ and $\hbox {DTW}_I$ cannot be judged by AUROC, since they do not provide probabilities with which to order the instances. AUROC demonstrates that the top three algorithms in terms of accuracy are significantly better than all others at ordering the data. We also compared all classifiers using a paired Student’s *t*-test instead of Wilcoxon sign rank test. For $\alpha =0.05$, there would only be two different decisions: STC is not significantly worse than ROCKET with a *t*-test, but is with a sign rank test; and CBOSS is signifacntly worse than STC with a sign rank test, but not with a *t*-test. Critical difference diagrams can sometimes mask differences between individual classifiers, because of the nature of forming cliques. It is worthwhile, therefore, presenting *p*-values and summarising accuracy distributions. Table 5 presents the pairwise *p*-values for all 11 combinations, with the upper diagonal being sign rank and the lower diagonal the *t*-test. Note that no adjustments for multiple testing have been made. The top clique using *t*-test would now include STC, but there are few practical differences. The differences in accuracy between the complete classifiers and $\hbox {DTW}_D$ are summarised in Fig. 8. Here we can see some of the wide spread of relative performances by classifiers over the datasets. STC has the widest distribution of difference in accuracies which explains the fact that STC has the biggest difference in test results between sign rank and *t*-test shown in Table 5.

**Table 5:** *P*-values for pairwise tests between classifiers

	RCKT	HC	CIF	ResNet	STC	$\hbox {DTW}_D$	gRSF	TSF	CBOSS	RISE	$\hbox {DTW}_I$
RCKT	0.0000	**0**.**1742**	0.5506	0.0619	**0**.**0283**	0.0004	0.0004	0.0039	0.0006	0.0024	0.0000
HC	**0**.**9128**	0.0000	0.5338	0.1285	0.0585	0.0092	0.0023	0.0004	0.0000	0.0003	0.0000
CIF	0.6660	0.6402	0.0000	0.2692	0.0520	0.0043	0.0009	0.0006	0.0017	0.0001	0.0001
ResNet	0.0759	0.1246	0.1184	0.0000	0.9899	0.3282	0.5812	0.5506	0.2277	0.1500	0.0092
STC	**0**.**4282**	0.0621	0.0580	0.4512	0.0000	0.1068	0.1513	0.0578	**0**.**0074**	0.0020	0.0004
$\hbox {DTW}_D$	0.0005	0.0159	0.0166	0.2989	0.1630	0.0000	0.4091	0.8689	0.7509	0.6204	0.0022
gRSF	0.0003	0.0168	0.0041	0.7645	0.1532	0.5509	0.0000	0.5338	0.5338	0.1218	0.0010
TSF	0.0039	0.0044	0.0006	0.6544	0.0837	0.7847	0.7881	0.0000	0.6938	0.5338	0.0230
CBOSS	0.0008	0.0022	0.0037	0.3621	**0**.**0669**	0.9258	0.5020	0.7520	0.0000	0.3949	0.0054
RISE	0.0024	0.0008	0.0000	0.3258	0.0044	0.6638	0.1534	0.3549	0.4838	0.0000	0.1307
$\hbox {DTW}_I$	0.0000	0.0001	0.0001	0.0036	0.0009	0.0074	0.0028	0.0096	0.0042	0.1013	0.0000

The upper diagonal values are found using the Wilcoxon sign-rank test. The lower diagonal are found using a paired *t*-test. So, for example the *p*-value for STC vs BOSS is 0.0074 using a sign rank test, but 0.0669 with paired *t*-test. Classifiers are ordered by overall rank, so a *p*-value below the critical value for STC vs BOSS indicates STC is significantly more accurate on these data

**Fig. 9:**
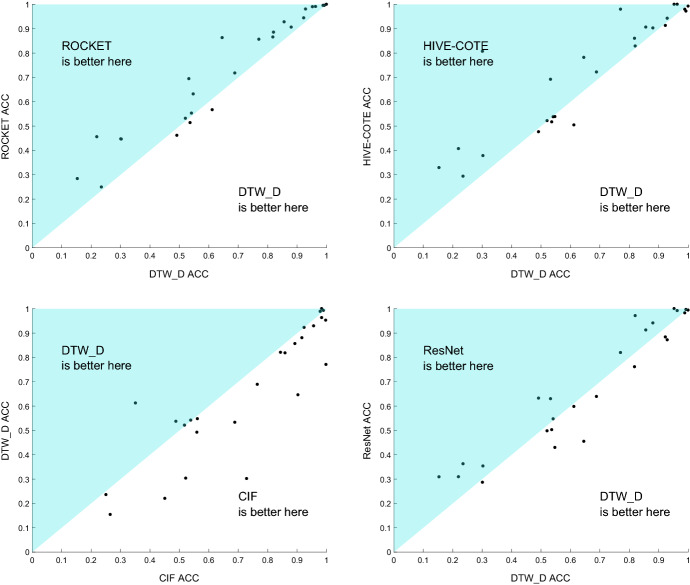
Scatter plots of the accuracy of ROCKET, HIVE-COTE, CIF, and ResNet against $\hbox {DTW}_D$

Figure 9 demonstrates this further for the top clique of classifiers, and shows scatter plots of test accuracies against the $\hbox {DTW}_D$ benchmark. ROCKET is better on 22 and worse on 3, with a mean difference of 5.9% and median difference of 3.3%. HIVE-COTE is better on 17 and worse on 9, with a mean difference of 5.8% and median difference of 3.28%. CIF is better on 19 and worse on 7 with a mean difference of 6.5% and a median difference of 3.18%. ResNet is better on 14 and worse on 12 with a mean difference of 1.7% and a median difference of 0.45%.

Table 6 gives the detailed results for the three classifiers significantly better than $\hbox {DTW}_D$, including the standard error over resamples. This table demonstrates that there will still be problems, such as HandWriting, where $\hbox {DTW}_D$ is the best approach but that, lacking any extra information, the other algorithms will generally give significantly more accurate classifiers. While ROCKET and HIVE-COTE lose by a relatively smaller margin when $\hbox {DTW}_D$ does outperform them, the HandWriting case shows that CIF has a much clearer gap in the types of problem it can effectively handle.

**Table 6:** Average accuracies with standard error over re-samples for $\hbox {DTW}_D$ and the three classifiers significantly more accurate than $\hbox {DTW}_D$

	$\hbox {DTW}_D$ (%)	ROCKET (%)	CIF (%)	HIVE-COTE (%)
AWR	$98.87 {\pm }0.05$	$\mathbf{99.56 }{\pm }{} \mathbf{0.13 }$	$97.89 {\pm }0.15$	$97.99 {\pm }0.10$
AF	$23.56 {\pm }1.39$	$24.89 {\pm }1.68$	$25.11 {\pm }2.18$	$\mathbf{29.33 }{\pm }{} \mathbf{1.31 }$
BM	$95.25 {\pm }0.23$	$99.00 {\pm }0.00$	$99.75 {\pm }0.14$	$\mathbf{100.0 }{\pm }{} \mathbf{0.84 }$
CR	$\mathbf{100.0 }{\pm }{} \mathbf{0.00 }$	$\mathbf{100.0 }{\pm }{} \mathbf{0.13}$	$98.38 {\pm }0.29$	$99.26 {\pm }0.00$
DDG	$49.20 {\pm }0.99$	$46.13 {\pm }1.04$	$\mathbf{56.00 }{\pm }{} \mathbf{1.03 }$	$47.60 {\pm }1.20$
EW	$64.58 {\pm }0.53$	$86.28 {\pm }1.21$	$\mathbf{90.33 }{\pm }{} \mathbf{0.54 }$	$78.17 {\pm }0.62$
EP	$96.30 {\pm }0.17$	$99.08 {\pm }0.00$	$98.38 {\pm }0.27$	$\mathbf{100.0 }{\pm }{} \mathbf{0.26 }$
EC	$30.15 {\pm }0.54$	$44.68 {\pm }0.43$	$72.89 {\pm }0.56$	$\mathbf{80.68 }{\pm }{} \mathbf{0.50 }$
ER	$92.91 {\pm }0.12$	$\mathbf {98.05 }{\pm }{} \mathbf{0.49 }$	$95.65 {\pm }0.42$	$94.26 {\pm }0.40$
FD	$53.28 {\pm }0.23$	$\mathbf {69.42 }{\pm }{} \mathbf{0.30 }$	$68.89 {\pm }0.27$	$69.17 {\pm }0.14$
FM	$54.17 {\pm }0.90$	$\mathbf {55.27 }{\pm }{} \mathbf{0.84 }$	$53.90 {\pm }0.81$	$53.77 {\pm }0.93$
HMD	$30.32 {\pm }1.00$	$44.59 {\pm }0.87$	$\mathbf{52.21 }{\pm }{} \mathbf{1.08 }$	$37.79 {\pm }0.81$
HW	$\mathbf{61.21 }{\pm }{} \mathbf{0.42 }$	$56.67 {\pm }0.42$	$35.13 {\pm }0.40$	$50.41 {\pm }0.42$
HB	$68.88 {\pm }0.37$	$71.76 {\pm }0.02$	$\mathbf{76.52 }{\pm }{} \mathbf{0.30 }$	$72.18 {\pm }0.52$
LIB	$88.04 {\pm }0.44$	$90.61 {\pm }0.45$	$\mathbf{91.67 }{\pm }{} \mathbf{0.49 }$	$90.28 {\pm }0.61$
LSST	$54.76 {\pm }0.08$	$\mathbf{63.15 }{\pm }{} \mathbf{0.16 }$	$56.17 {\pm }0.22$	$53.84 {\pm }0.14$
MI	$52.10 {\pm }0.73$	$\mathbf{53.13 }{\pm }{} \mathbf{0.78 }$	$51.80 {\pm }1.03$	$52.17 {\pm }0.74$
NATO	$82.04 {\pm }0.32$	$\mathbf{88.54 }{\pm }{} \mathbf{0.44 }$	$84.41 {\pm }0.32$	$82.85 {\pm }0.32$
PD	$99.28 {\pm }0.05$	$\mathbf{99.56 }{\pm }{} \mathbf{0.14 }$	$98.97 {\pm }0.08$	$97.19 {\pm }0.06$
PEMS	$77.05 {\pm }0.58$	$85.63 {\pm }0.38$	$\mathbf{99.85 }{\pm }{} \mathbf{0.09 }$	$97.98 {\pm }0.59$
PS	$15.39 {\pm }0.10$	$28.35 {\pm }0.12$	$26.56 {\pm }0.13$	$\mathbf{32.87 }{\pm }{} \mathbf{0.07 }$
RS	$85.64 {\pm }0.26$	$\mathbf{92.79 }{\pm }{} \mathbf{0.45 }$	$89.30 {\pm }0.51$	$90.64 {\pm }0.37$
SRS1	$81.81 {\pm }0.35$	$\mathbf{86.55 }{\pm }{} \mathbf{0.31 }$	$85.94 {\pm }0.28$	$86.02 {\pm }0.32$
SRS2	$\mathbf{53.69 }{\pm }{} \mathbf{0.49 }$	$51.35 {\pm }0.59$	$48.87 {\pm }0.56$	$51.67 {\pm }0.67$
SWJ	$22.00 {\pm }1.87$	$\mathbf{45.56 }{\pm }{} \mathbf{2.72 }$	$45.11 {\pm }2.65$	$40.67 {\pm }1.54$
UW	$92.28 {\pm }0.21$	$\mathbf{94.43 }{\pm }{} \mathbf{0.35 }$	$92.42 {\pm }0.32$	$91.31 {\pm }0.23$

**Fig. 10:**
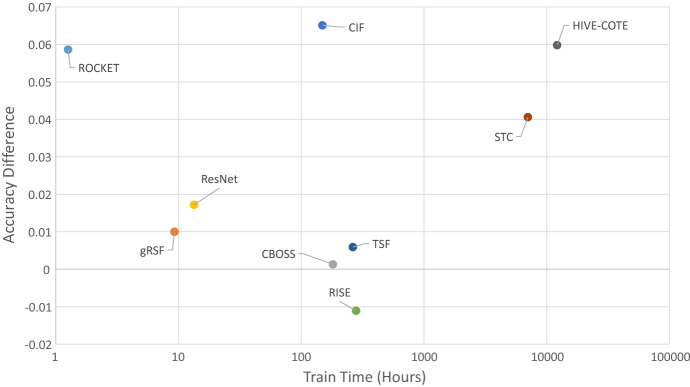
Average difference in accuracy to $\hbox {DTW}_D$ versus train time for 9 MTSC algorithms

Run times are hard to compare because of both software and hardware differences. Nevertheless, to get some idea of the relative performance, we recorded run time for all experiments. Table 7 gives the summary run time information, and Fig. 10 plots accuracy against runtime.

**Table 7:** Total run time for a single re-sample of all 26 problems and mean difference in accuracy to $\hbox {DTW}_D$ for 9 classifiers

Classifier	Total time (h)	Difference in accuracy to $\hbox {DTW}_D$ (%)
ROCKET	1.26	5.86
gRSF	9.27	1.0
ResNet	13.38	1.72
CIF	148.55	6.51
CBOSS	181.60	0.13
TSF	263.88	0.59
RISE	279.64	$-$ 1.11
STC	7019.69	4.06
HIVE-COTE	12,172.44	5.98

ROCKET lives up to its name: it is by far the fastest algorithm and remarkably can build a model for all 26 data sets in just over an hour. If it is set to be threaded, it completes 30 re-samples of the 26 problems in less than 2 h. Given its accuracy, this seems strong evidence to support it as a new baseline. CIF is much slower, requiring about 6 days for all the problems, but it averages around 5 h per problem. HIVE-COTE is by far the slowest and if run sequentially would take over a year to complete all the problems. Strictly speaking, it would violate our run time constraints if we ran it in this way. However, we include it here because we did not run it sequentially. We ran each component/dimension combination independently and in parallel. The nature of dimension independent ensembles makes this much easier to do than with algorithms such as MUSE and TapNet. It is also noteworthy that STC is the slowest component, but that is due to our parameter choice. STC is contracted, and defaults to 24 h compute time on each dimension. For high dimensional problems, this would lead to huge run times if completed sequentially. However, it is hardly ever necessary to search for shapelets for 24 h. Table 7 shows that, on average, STC is 4.06% more accurate than DTW, but overall, it is not significantly better. This demonstrates that there are problems where a specific representation is much better.

Memory usage is even harder to determine, since we are concerned with the maximum memory used during a run, not just the final memory footprint of the classifier. We can record the maximum memory usage in tsml, but this capability is not yet in sktime and its variants. Table 8 shows the maximum and total memory usage of eight tsml classifiers. HIVE-COTE is the most memory intensive classifier, but even HIVE-COTE required at most 3.5 GB (MotorImagery) and just 21 GB for all problems. Memory is not a significant constraint for these classifiers.

**Table 8:** Memory usage (in MB) for eight tsml classifiers

Classifier	Max memory	Total memory
$\hbox {DTW}_I$	1883	5587
$\hbox {DTW}_D$	1845	5952
RISE	2624	10,242
TSF	2670	10,632
CBOSS	2675	10,537
STC	2163	9778
CIF	2954	15,900
HIVE-COTE	3577	21,217

Max memory is the maximum memory on any single problem, total memory is the agregated memory over all twenty six problems

**Fig. 11:**
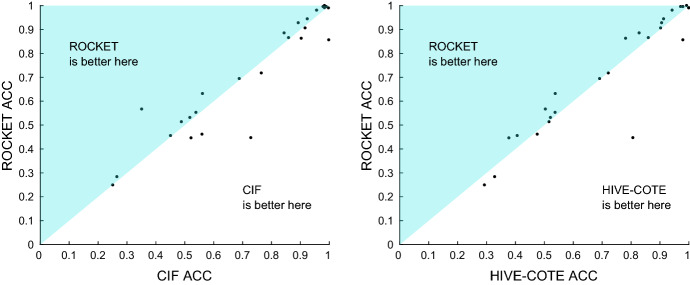
Scatter plots of accuracy on 26 UEA MTSC problems for ROCKET against CIF and HIVE-COTE. ROCKET beats CIF on 17 problems, with mean and median difference in accuracy are $-0.12\%$ and 0.85%). ROCKET beats HIVE-COTE on 17 problems with mean and median difference in accuracy are $-0.66\%$ and 0.66%

To summarise, only three of the ten classifiers able to complete on all problems are significantly more accurate than the baseline $\hbox {DTW}_D$ (ROCKET, CIF and HIVE-COTE). Figure 11 shows the relative performance of ROCKET against CIF and HIVE-COTE. These figures show that ROCKET consistently beats the other two, but that when it fails, it tends to fail badly. This is demonstrated by the fact it is marginally worse on average than both when looking at the mean difference, but better when the median is considered. It is also highlighted with the *p*-values shown in Table 5. The non-parametric sign rank test *p*-values for ROCKET against CIF and HIVE-COTE are much lower than the parametric *t*-test *p*-values. ROCKET performs at least as well as HIVE-COTE and CIF and is by far the fastest, and would be our recommended starting point for an investigation of a new MTSC problem. The evidence of the occasional large failure could help drive future design improvements.

### Comparison of sixteen classifiers on twenty datasets

**Fig. 12:**
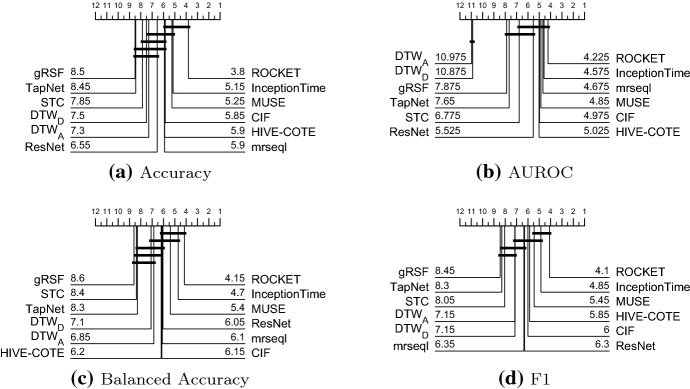
Critical difference diagrams for the top 12 classifiers on the 20 equal length UEA datasets all algorithms completed

$\hbox {DTW}_A$, MUSE, MrSEQL, TapNet and InceptionTime did not complete on all problems. Rather than a lengthy individual analysis, we present the results for the twenty problems which all algorithms completed. For clarity, we remove the four worst performing classifiers ($\hbox {DTW}_I$, RISE, TSF and CBOSS). Figure 12 shows the critical difference diagrams for the top twelve classifiers on the twenty data sets that all algorithms completed within our constraints.

MUSE is memory intensive. On these 20 problems it required an average of 8 GB, compared to just 500 MB for HIVE-COTE. Fewer datasets make it harder to detect significant differences. The top clique is now (ROCKET, InceptionTime, MUSE, CIF). However, these cliques do not reflect the differences to the baseline. With a critical value of $\alpha =0.05$, only ROCKET and CIF are significantly better than $\hbox {DTW}_D$ on these 20 problems. With 25 problems, InceptionTime is also significantly better than $\hbox {DTW}_D$, as is HIVE-COTE with 26. Table 9 gives the *p*-value for the pairwise test on the datasets completed by each algorithm.

**Table 9:** Performance relative to the benchmark classifier $\hbox {DTW}_D$

Algorithm	Completed data	*P*-value	W/D/L
MUSE	20	0.1005	13/0/7
TapNet	23	0.9015	10/0/13
MrSEQL	24	0.0593	16/0/8
$\hbox {DTW}_A$	25	0.6900	10/2/13
InceptionTime	25	0.0149	17/0/8
STC	26	0.1067	15/0/11
HIVE-COTE	26	0.0043	17/0/9
CIF	26	0.0092	19/0/7
ROCKET	26	0.0004	22/1/3

*P*-value is from the Wilcoxon sign rank test

MUSE does well, but is so memory intensive it will be hard to use for many problems. MrSEQL is also promising, although not significantly better than DTW. It is not clear why it failed to complete the two problems. InceptionTime, HIVE-COTE and CIF all beat the baseline and have potential. However, the stand out classifier is still ROCKET. It has the lowest average rank, beats the baseline on the most problems and it is incredibly quick, especially when parallelised. We think it is the clear winner of this experimental study.

### Analysis by problem

Table 11 shows the average test accuracy by problem and Table 10 shows the performance of the best classifier on each problem. Some problems are trivial, with most algorithms getting close to perfect results. These are: BasicMotions (BM); Cricket (CR); and Epilepsy (EP). Conversely, with other problems classifiers rarely do better than random guessing. We would put Heartbeat (HB) and SelfRegulationSCP2 (SRS2) in this category.

On other problems, the majority of classifiers find little or no information, but one or more algorithms fare much better. With AtrialFibrillation (AF), most classifiers are no better than random guessing (25%) and are worse than predicting the majority class (33%). MUSE alone finds useful information with an accuracy of 74%. The best algorithm for EthanolConcentration (EC) is STC, with an accuracy of 82%, whereas the three deep learning algorithms do little better than random guessing. These results demonstrate the importance of the representation for time series classification and highlight the need to find the right tool for the job.

Table 10 highlights another feature not immediately apparent from the aggregated results. The three deep learning algorithms TapNet, ResNet and InceptionTime (IT) are the most accurate approach on 9 of the problems. Neural networks tend to have high variance over problems, often either doing very well or very poorly on a particular problem. Received wisdom would suggest that beyond any data-driven characteristics that favour particular representations, deep learning approaches would have a comparative advantage (or disadvantage) on problems with more (less) training cases. This would appear to hold weight in the cases of FaceDetection (5890 cases), PenDigits (7494 cases) and PhonemeSpectra (3315 cases), but none of the others where a deep learning approach wins are large datasets. In particular, InceptionTime wins on DuckDuckGeese, which only has 50 train cases but 1345 dimensions and 270 time points. Here, it is likely the bottleneck operation that is successfully stripping down the large and sparse spectogram dimension space while other classifiers are less able to find sufficiently clean features.

**Table 10:** Best classifiers by problem

Problem	Classes	Default (%)	$\hbox {DTW}_D$ (%)	Best (%)	Algorithm(s)
AWR	25	4.00	98.87	99.56	ROCKET
AF	3	33.3	23.56	74.00	MUSE
BM	4	25.0	95.25	100.0	IT, MUSE, HC, ResNet, gRSF, RISE
CR	12	8.33	100.0	100.0	ROCKET, $\hbox {DTW}_A$, $\hbox {DTW}_D$
DDG	5	20.0	49.20	63.47	IT
EW	5	42.0	64.58	90.33	CIF
EP	4	26.8	96.30	100.0	HC
EC	4	25.1	30.15	82.36	STC
ER	6	16.7	92.91	98.05	ROCKET
FD	2	50.0	53.28	77.24	IT
FM	2	50.0	54.17	56.13	IT
HMD	4	18.9	30.32	52.21	CIF
HW	26	3.8	61.21	65.74	IT
HB	2	72.2	68.88	76.52	CIF
LIB	15	6.7	88.04	94.11	ResNet
LSST	14	31.5	54.76	63.62	MUSE
MI	2	50.0	52.10	53.80	TSF
NATO	6	16.7	82.04	97.11	ResNet
PD	10	10.4	99.28	99.68	IT
PEMS	7	11.6	77.05	99.85	CIF
PS	39	2.6	15.39	36.74	IT
RS	4	28.3	85.64	92.79	ROCKET
SRS1	2	50.2	81.81	95.68	TapNet
SRS2	2	50.0	53.69	53.69	$\hbox {DTW}_D$
SWJ	3	33.3	22.00	45.56	ROCKET
UW	8	12.5	92.28	94.43	ROCKET

### Explanatory analysis case studies

Ultimately, we would like to form a rationale for why one algorithm does better than another on a specific dataset. This would help improve our understanding of when to use one approach over another, and would guide future algorithm development. However, given the complexity of some of these algorithms, this is non trivial. We examine two datasets where there is a wide variance in algorithm performance and attempt to explain the characteristics of the data that confound certain classifiers.

#### Ethanol concentration

**Fig. 13:**
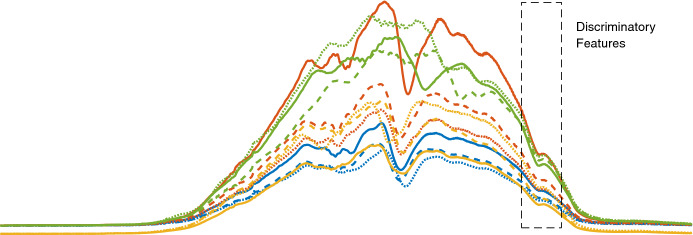
Visualisation of the first instance of each class by colour for the EthanolConcentration data, with dimension separated by line pattern

Ethanol Concentration has a wide range of performance results. The deep learning approaches and the DTW variants do little better than random guessing. CIF, however, achieves accuracy over 70% and STC gets over 80% accuracy. The key confounding aspect of this data is that the discriminatory features lie only in a small region of the series, and that the variation in this small region that allows the detection of the class is much lower than the variation in the rest of the spectra, which is class independent. CIF and STC are designed to mitigate this problem. CIF selects random intervals, whereas STC finds phase independent subseries. A sample of series from each class is shown in Fig. 13, with the discriminatory interval marked.

**Fig. 14:**
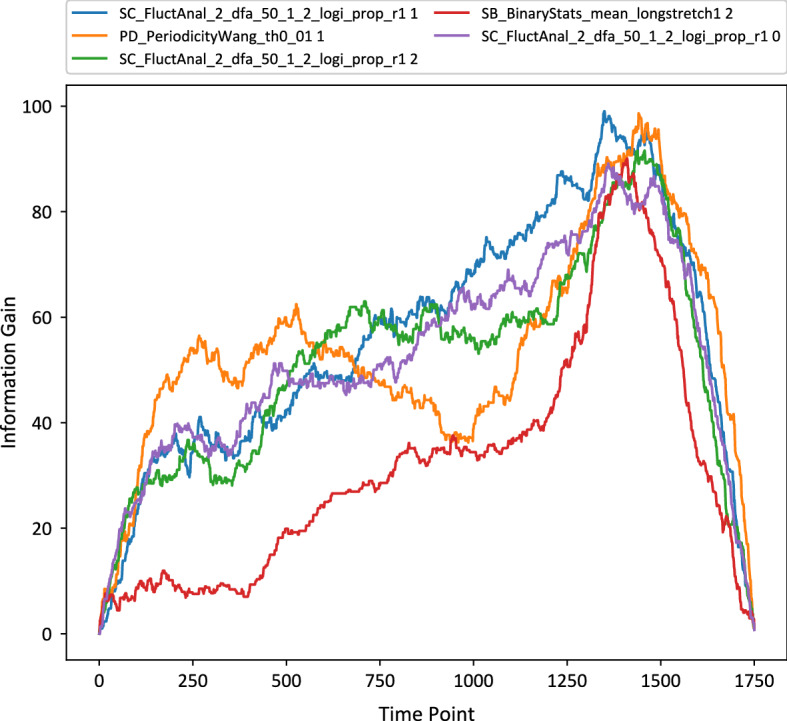
Temporal importance diagram generated by a CIF with no attribute subsampling on the EthanolConcentration data. Legend displays the CIF feature followed by dimension index

We take a deeper look into how both CIF and InceptionTime made their classifications using visualisation techniques for each. For CIF we can adapt the visualisation mechanism (Middlehurst et al. 2020) for the multivariate case. We train a separate CIF model with no attribute subsampling to allow for important feature and interval combinations to be chosen more often. Figure 14 shows the CIF temporal importance curves for the EthanolConcentration data. As shown the majority of information gain in nodes throughout the forest is concentrated around the discriminatory interval. A single fluctuation analysis feature appears as one of the top features for all 3 dimensions. Using CIF we can also estimate the importance of individual dimensions by looking at the number of times each dimensions is used in decision tree nodes. The 3 dimensions for EthanolConcentration have near identical importance, with each occurring in just over 8000 nodes throughout the forest. While this shows that each dimension contains useful information, it does not necessarily discount redundancy between them.

**Fig. 15:**
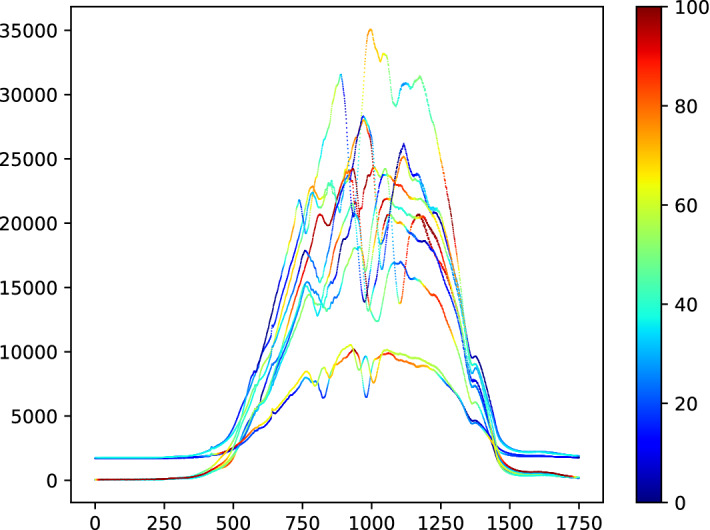
Class Activation Mappings for one network in the InceptionTime ensemble on 10 random test instances of EthanolConcentration (only one dimension of each instance shown for clarity)

Figure 15 displays the Class Activation Maps (CAM) (Wang et al. 2017; Fawaz et al. 2019) for a random selection of test cases of the EthanolConcentration data. These highlight temporal areas that highly contributed towards the network’s prediction of an instance. It demonstrates that InceptionTime could not reliably isolate the informative interval, and found spurious mappings from the high-noise area of the series to the labels. InceptionTime is an ensemble of five networks with different random initialisations. All five have similar looking CAM representations.

**Fig. 16:**
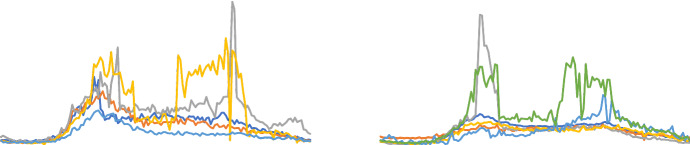
Visualisation of two sets of five dimensions for the PEMS-SF data

**Fig. 17:**
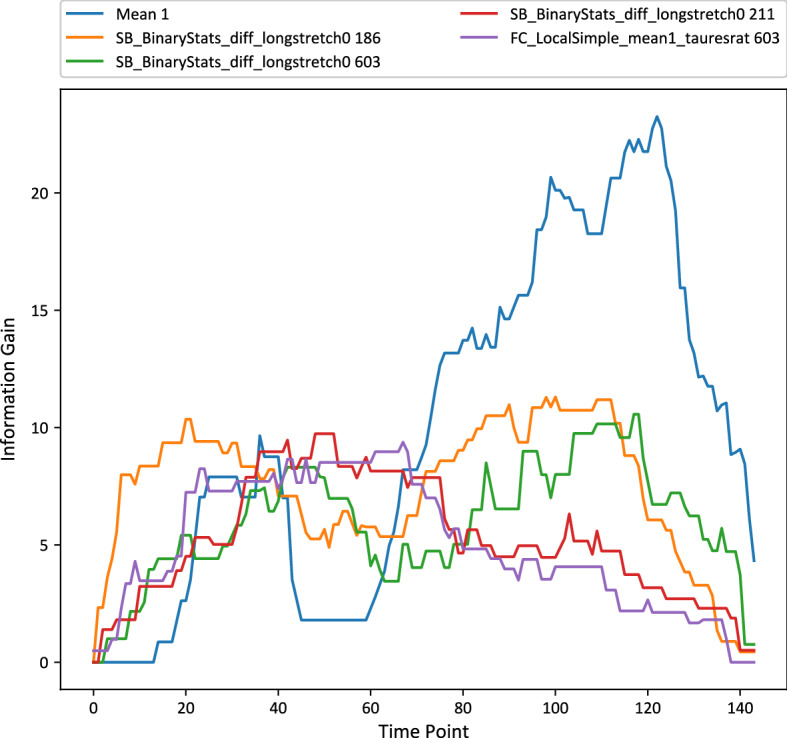
Temporal importance diagram generated by a CIF with no attribute subsampling on the PEMS-SF data. Legend displays the CIF feature followed by dimension index

#### PEMS-SF

PEMS-SF describes the occupancy rate, between 0 and 1, of different car lanes of San Francisco bay area freeways from 963 different sensors (dimensions). The problem is to predict the correct day of the week. There is a wide range of performance on this problem. The three DTW approaches do worst (77–80%) and the three deep learning algorithms do not do much better (InceptionTime and ResNet (79–83%). Conversely, the interval techniques (CIF, TSF and RISE) and the shapelet based algorithms (MrSEQL, STC a gRFS) all achieve over 90% and CIF is almost perfect with an average accuracy of 99.84% and 100% accuracy on the default split. We suspect that the confounding factor here is the number of dimensions. To explore this hypothesis, we extract a case from the default test set where CIF gets the prediction correct. The case is the second data in the test data set. The correct class is Tuesday (class value 1). We cannot sensibly visualise all 963 dimensions. Figure 16 plots the two sets of five dimensions. The data highlights another possible important characteristic: the morning and afternoon rush hour peaks. It is possible that rush hour is much more discriminatory for day of the week, and algorithms that can discard the less important and possibly confounding periods do better. It is also possible that different times of the day are important for different dimensions. This case is predicted incorrectly as Thursday by InceptionTime, whereas both CIF and STC predict it correctly. InceptionTime thinks Tuesday is very unlikely. It assigns Tuesday a probability of 0.041 and Thursday 0.723. CIF is also very confident about its prediction: it assigns Tuesday a probability of 0.762. STC is less confident, in that it estimates the probability of Tuesday to be 0.305, but that is still the highest probability. So why are CIF and STC correct and InceptionTime wrong? Figure 17 shows the CIF temporal importance curves for the PEMS-SF data. Three of the curves (blue, orange and green) peak at the beginning and end of the series, covering a period prior to the first rush hour and during the second. The remaining curve (red and purple) cover the first rush hour period. Curves from both of these groups share summary statistics, implying that the time interval importance may differ between dimensions. Looking at tree nodes for dimension importance shows a few are repeatedly chosen when they appear, with only nine dimensions having hundreds of nodes throughout the forest. On the other side 747 of the dimensions appear less than ten times, if at all. This ability to select dimension and time intervals is likely an important factor to the success of CIF on the dataset.

**Fig. 18:**
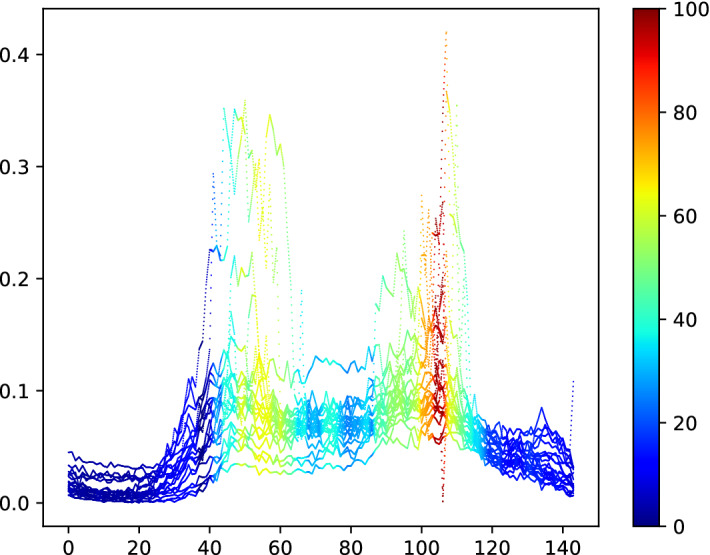
Class Activation Map for a network in InceptionTime on the examined case of PEMS-SF. Inception time predicted Thursday, while the true label was Tuesday. 20 Dimensions of the instance only are shown for clarity

Figure 18 shows the CAM of a network in InceptionTime for this problem case, highlighting the areas that lead to its prediction of Thursday. It seemingly correctly highlights the rush hour areas as important. However, in this case perhaps simply could not disambiguate between the different weekdays. Three of the networks in the ensemble strongly predicted Thursday, one predicted Wednesday with equal certainty, while the final member predicted Thursday while also giving Tuesday a probability of 0.153.

## Conclusions

This experimental analysis has demonstrated that MTSC is at an earlier stage of development than univariate TSC. The standard TSC benchmark, DTW, is still hard to beat and competitive with many more recently proposed alternatives. HIVE-COTE (with components all built independently on each dimension), CIF, ROCKET and InceptionTime are significantly better than DTW. Both CIF and ROCKET use some form of dimension dependent feature extraction. The performance of CIF, an improvement of the HIVE-COTE component TSF, suggests that introducing bespoke multivariate algorithms to the other HIVE-COTE components will improve overall performance. InceptionTime is the top performing algorithm on more problems than any other algorithm and should be the starting point for future work with neural networks. However, the real winner of this experimental analysis is ROCKET. We did not originally include ROCKET in this study (an early iteration of this paper is available on ArXiv (Pasos-Ruiz et al. 2020)) because multivariate capability is listed as future work in the related publication (Dempster et al. 2020). However, the authors of ROCKET contributed their code to the sktime toolkit with multivariate functionality, so we could include it in the study. This highlights the benefits of code sharing within a common framework.

ROCKET is the best ranked and by far the fastest classifier and would be our recommendation as the default choice for MTSC problems. Clearly, results on 26 data sets do not generalise to all problems. No algorithm will outperform all others all the time, and there is a place for a toolkit of approaches for the practitioner to drawn from. However, benchmarking is important, particularly when assessing new algorithms. Hence, we suggest that new algorithms in this domain be compared to $\hbox {DTW}_D$ and ROCKET as benchmarks. We make all results and the code to generate the results publicly available on the associated website and are happy to work with other researchers to help move the field forward and find new applications. The UEA MSTC archive is fairly new and needs more development. New data are being added and donations are always welcome. An expanded version with at least 50 data sets is planned for 2021. These experiments represent a platform for future development. We would expect that, in the near future, algorithms that explicitly model interactions between dimensions would outperform all of the algorithms presented here and advance the research field of MTSC.

## Appendix

See the Table 11.

**Table 11:** Accuracy of twelve algorithms averaged over thirty stratified re-samples data sets for the UEA MTSC archive

Problem	Default (%)	ROCKET (%)	IT (%)	MUSE (%)	CIF (%)	HC (%)	mrseql (%)	ResNet (%)	$\hbox {DTW}_A$ (%)
AWR	4.00	99.56	99.10	98.87	97.89	97.99	98.98	98.26	98.94
AF	33.3	24.89	22.00	74.00	25.11	29.33	36.89	36.22	22.44
BM	25.0	99.00	100.0	100.0	99.75	100.0	94.83	100.0	99.92
CR	8.33	100.0	99.44	99.77	98.38	99.26	99.21	99.40	100.0
DDG	20.0	46.13	63.47		56.00	47.60	39.27	63.20	56.67
EW	42.0	86.28			90.33	78.17	72.16	45.45	
EP	26.8	99.08	98.65	99.64	98.38	100.0	99.93	99.18	97.37
EC	25.1	44.68	27.92	48.64	72.89	80.68	60.18	28.62	29.57
ER	16.7	98.05	92.10	96.89	95.65	94.26	93.19	87.19	92.89
FD	50.0	69.42	77.24		68.89	69.17		62.97	53.13
FM	49.0	55.27	56.13	54.77	53.90	53.77	55.53	54.70	54.93
HMD	18.9	44.59	42.39	38.02	52.21	37.79	35.23	35.32	30.72
HW	3.8	56.67	65.74	51.85	35.13	50.41	54.04	59.78	60.55
HB	72.2	71.76	73.20	73.59	76.52	72.18	72.52	63.89	68.07
LIB	6.7	90.61	88.72	90.30	91.67	90.28	86.57	94.11	87.85
LSST	31.5	63.15	33.97	63.62	56.17	53.84	60.28	42.94	56.96
MI	50.0	53.13	51.17		51.80	52.17	53.00	49.77	50.37
NATO	16.7	88.54	96.63	87.13	84.41	82.85	86.43	97.11	81.48
PD	10.4	99.56	99.68	98.68	98.97	97.19	97.14	99.64	99.27
PEMS	11.6	85.63	82.83		99.85	97.98	97.15	81.95	78.73
PS	2.6	28.35	36.74		26.56	32.87		30.86	15.39
RS	28.3	92.79	91.69	89.56	89.30	90.64	88.73	91.23	85.79
SRS1	50.2	86.55	84.69	73.58	85.94	86.02	82.86	76.11	81.34
SRS2	50.0	51.35	52.04	49.52	48.87	51.67	49.61	50.24	52.43
SWJ	33.3	45.56	42.00	34.67	45.11	40.67	42.00	30.89	25.56
UW	12.5	94.43	91.23	90.39	92.42	91.31	91.32	88.35	91.51
AWR	97.51	98.87	97.13	98.21	94.82	97.56	95.73	94.31	
AF	31.78	23.56	30.22	27.56	29.78	30.44	24.44	34.67	
BM	97.92	95.25	99.17	100.00	99.83	98.75	100.0	72.17	
CR	98.94	100.0	97.50	97.41	93.15	97.55	97.78	95.74	
DDG	43.47	49.20	58.27	44.47	38.87	43.07	50.80	29.27	
EW	74.68	64.58		83.00	76.62	62.80	81.93	44.20	
EP	98.74	96.30	96.09	96.01	97.34	99.83	99.86	67.03	
EC	82.36	30.15	28.99	34.06	45.42	39.62	49.16	30.68	
ER	84.28	92.91	89.46	91.98	89.84	84.48	82.44	91.42	
FD	69.76	53.28	52.87	55.36	68.95	52.32	51.17	51.53	
FM	53.40	54.17	51.33	54.43	53.17	51.03	52.10	55.50	
HMD	34.95	30.32	32.34	32.07	48.51	28.87	28.24	26.67	
HW	28.77	61.21	32.95	36.96	36.42	49.09	18.27	34.33	
HB	72.15	68.88	73.97	74.89	72.28	72.15	73.22	63.80	
LIB	84.46	88.04	83.63	75.56	79.72	85.26	81.67	78.63	
LSST	57.82	54.76	46.33	58.15	34.31	43.62	50.58	49.57	
MI	50.83	52.10		51.87	53.80	52.37	49.83	49.63	
NATO	84.35	82.04	90.30	82.37	77.72	82.48	80.59	76.07	
PD	97.70	99.28	93.65	96.12	94.11	95.61	87.47	99.22	
PEMS	98.40	77.05	79.21	91.27	96.76	96.57	98.98	80.23	
PS	30.62	15.39		22.71	14.52	19.43	26.78	10.18	
RS	88.09	85.64	85.81	87.79	88.29	88.90	84.17	81.69	
SRS1	84.73	81.81	95.68	79.74	84.73	81.33	73.17	80.63	
SRS2	51.63	53.69	53.46	48.69	50.65	50.02	50.28	48.48	
SWJ	44.00	22.00	35.11	38.44	33.33	36.89	34.00	35.78	
UW	87.03	92.28	88.39	89.59	85.05	86.13	71.11	87.58	

Default accuracy is for predicting the majority class

## Footnotes

1 www.timeseriesclassification.com/mtsc_bakeoff.php.

2 https://github.com/alan-turing-institute/sktime/blob/master/sktime/transformers/series_as_features/rocket.py.

3 https://github.com/alan-turing-institute/sktime/blob/master/sktime/classification/shapelet_based/mrseql/mrseql.pyx.

4 http://www.mustafabaydogan.com/.

5 http://www.mustafabaydogan.com/multivariate-time-series-discretization-for-classification.html.

6 https://www.timeseriesclassification.com/.

7 https://www.kaggle.com/c/decoding-the-human-brain/data.

8 http://bbci.de/competition/iv/.

9 https://aaltd16.irisa.fr/challenge/.

10 https://www.xenocanto.org.

11 https://www.physionet.org/physiobank/database/challenge/2016/.

12 https://plasticc.org/.

13 www.pems.dot.ca.gov.

14 https://github.com/alan-turing-institute/sktime.

15 https://github.com/uea-machine-learning/tsml.

16 https://github.com/sktime/sktime-shapelets-forest.
